# Supplementary material for: The diagnostic application of RNA sequencing in patients with thyroid cancer: an analysis of 851 variants and 133 fusions in 524 genes
Source: BMC Bioinformatics. 2016 Jan 11;17(Suppl 1):6. doi: 10.1186/s12859-015-0849-9 (PMC4895782; doi:10.1186/s12859-015-0849-9)
Supplement: Additional file 1: — Sequencing quality metrics for 851 variants. (PDF 49 kb) [file 12859_2015_849_MOESM1_ESM.pdf]

Additional file 1. Sequencing quality metrics for 851 variants.

Reads	Median (± s.d.)
Total paired reads	48,312,088 (± 27,931,712)
Aligned paired reads	37,332,723 (± 20,455,280)
% of variants (per genomic location) covered to:	Min-Max 90th Percentile Range[a]
≥x1	71.0 - 99.9
≥x5	56.0 – 98.0
≥x10	41.0 - 96.0
≥x20	24.0 - 93.0
≥x30	17.0 - 90.0
≥x100	7.0 - 75.0

[a]Coverage varies dependent on the subtype of the sample tested.
