# Supplementary figures and images for: The diagnostic application of RNA sequencing in patients with thyroid cancer: an analysis of 851 variants and 133 fusions in 524 genes
Source: BMC Bioinformatics. 2016 Jan 11;17(Suppl 1):6. doi: 10.1186/s12859-015-0849-9 (PMC4895782; doi:10.1186/s12859-015-0849-9)

## Variants (n=851) Arranged by Chromosomal Location

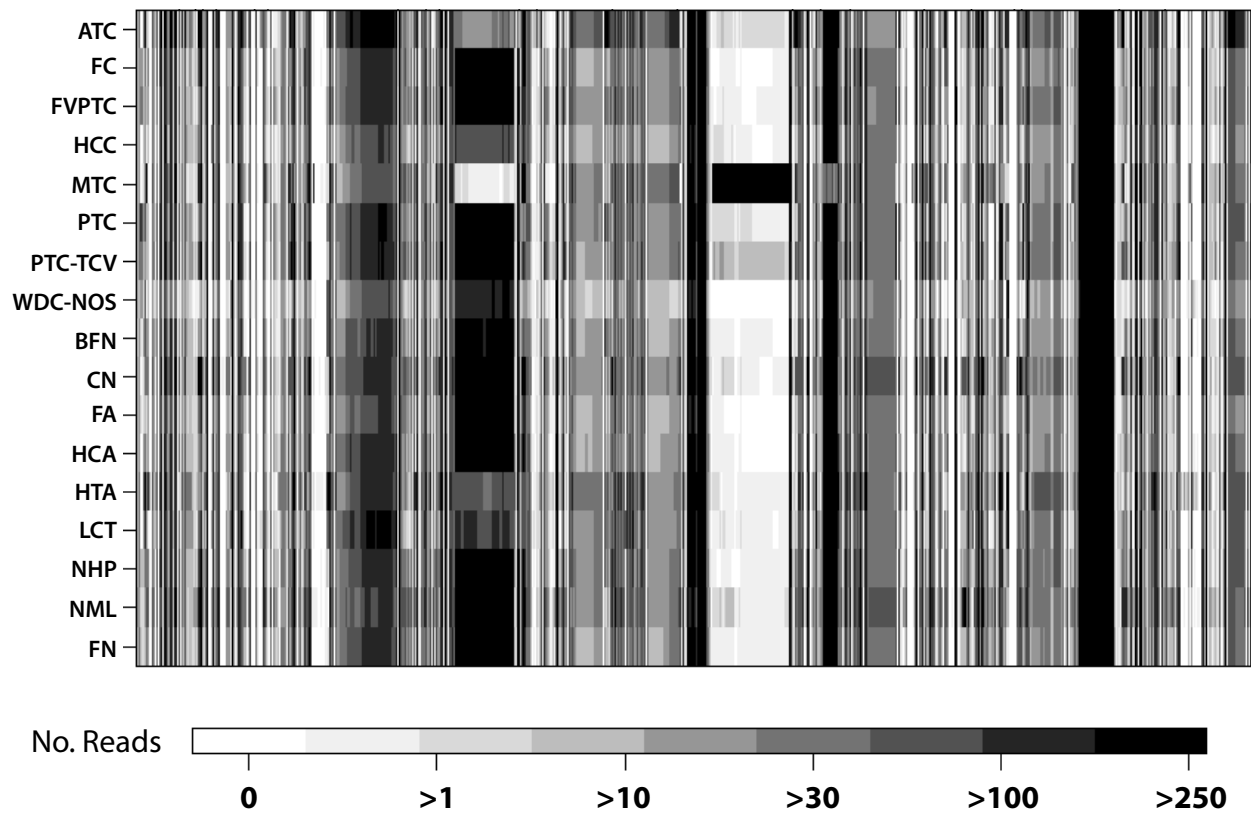

Supplement: Additional file 2: — Read-depths obtained per variant and per subtype. (PDF 393 kb) [file 12859_2015_849_MOESM2_ESM.pdf]
