# Supplementary material for: The diagnostic application of RNA sequencing in patients with thyroid cancer: an analysis of 851 variants and 133 fusions in 524 genes
Source: BMC Bioinformatics. 2016 Jan 11;17(Suppl 1):6. doi: 10.1186/s12859-015-0849-9 (PMC4895782; doi:10.1186/s12859-015-0849-9)
Supplement: Additional file 3: — Variants included in the panel ( n = 851). (PDF 596 kb) [file 12859_2015_849_MOESM3_ESM.pdf]

Supplemental Table 2. Variants detected using GATK or SamTools variant calling methods.

Gene	Amino acid change	Nucleotide Change	Genomic Variant ID	Note	Variant Detected By GATK	Variant Detected By Samtools
ABL1	p.E453K	c.1357G>A	9:133753888__G__A			
ABL2	p.S1080*	c.3239C>G	1:179077163__G__C			
ACVR2A	p.I458T	c.1373T>C	2:148684674__T__C			
ACVR2B	p.V359L	c.G1075C	3:38523689__G__C			
AKAP9	p.H2680R	c.8039A>G	7:91711855__A__G			Yes
AKAP9	p.R3668G	c.11002A>G	7:91730275__A__G			
AKT1	p.E133D	c.399G>C	14:105242025__C__G			
AKT1	p.E17K	c.49G>A	14:105246551__C__T			
AKT2	p.E17K	c.49G>A	19:40762959__C__T			
ALK	p.P693S	c.2077C>T	2:29474098__G__A			
ALPK2	p.I2145V	c.6433A>G	18:56149135__T__C			
ALPK2	p.L575P	c.1724T>C	18:56246284__A__G			
ALPK2	p.S164P	c.490T>C	18:56247518__A__G			
APC	p.D1714Y	c.5140G>T	5:112176431__G__T			
APC	p.R213*	c.637C>T	5:112116592__C__T			
APOL2	p.A238V	c.C713T	22:36623751__G__A			
ARID1B	p.D1723N	c.5167G>A	6:157527496__G__A			
ARID1B	p.Q1382*	c.4144C>T	6:157521926__C__T			
ARID1B	p.Q1657K	c.4969C>A	6:157525128__C__A			
ARID1B	p.R1935H	c.5804G>A	6:157528133__G__A			
ARID1B	p.S355fs	c.1239delG	6:157100301__CG__C			
ARID2	p.Q1031*	c.3091C>T	12:46244997__C__T			
ARID5B	p.K1027R	c.3080A>G	10:63852302__A__G			Yes
ASH1L	p.A93G	c.278C>G	1:155491033__G__C			
ASH1L	p.P100S	c.298C>T	1:155491013__G__A			
ASH1L	p.T2910A	c.8728A>G	1:155307970__T__C			
ASXL1	p.G1299D	c.3896G>A	20:31024411__G__A			
ATF6B	p.N701S	c.2102A>G	6:32083526__T__C			
ATM	p.L2132V	c.6394C>G	11:108190727__C__G			
ATM	p.L2452P	c.7355T>C	11:108200988__T__C			
ATM	p.T1908fs	c.5723delC	11:108178671__AC__A			
ATM	p.T2228I	c.6683C>T	11:108196147__C__T			
ATM	p.W2845C	c.8535G>T	11:108216586__G__T			
ATP1A1	p.V332G	c.995T>G	1:116932301__T__G			
ATR	p.L1013V	c.3037C>G	3:142268455__G__C			
ATR	p.Q1073*	c.3217C>T	3:142266707__G__A			
ATR	p.R993_splice	c.2977_splice	3:142268516__C__G			
ATRX	p.K299E	c.895A>G	X:76939853__T__C			
AXIN1	p.A330V	c.989C>T	16:364573__G__A			

Gene	Amino acid change	Nucleotide Change	Genomic Variant ID	Note	Variant Detected By GATK	Variant Detected By Samtools
AXIN2	p.R77W	c.229C>T	17:63554510__G__A			
AZGP1	p.I96M	c.288C>G	7:99569418__G__C			
BAP1	p.E31G	c.92A>G	3:52443600__T__C			
BCL11A	p.E611K	c.1831G>A	2:60688216__C__T			
BCL6	p.S597C	c.1790C>G	3:187443336__G__C			
BCL9	p.K86N	c.258G>T	1:147084886__G__T			
BCL9	p.R584I	c.1751G>T	1:147091712__G__T			
BCLAF1	p.N221I	c.662A>T	6:136599357__T__A			Yes
BCOR	p.D1226fs	c.3677_3678insA	X:39923030__A__AT			
BCOR	p.R1261G	c.3781A>G	X:39922927__T__C			Yes
BMP6	p.R379Q	c.1136G>A	6:7862663__G__A			Yes
BRAF	p.598_599insKIGDFGLA	c.1772_1795AAATAGGTGATTTTGGTCTAGCTA>AAATAGGTGATTTTGGTCTAGCTAAAATAGGTGATTTTGGTCTAGCTA	7:140453140__TTAGCTAGACCAAAATCACCTATTT__TTAGCTAGACCAAAATCACCTATTTTAGCTAGACCAAAATCACCTATTT			
BRAF	p.598_599insNIFLHEDLTVKIGDFGLA	c.1742_splice	7:140453140__TTAGCTAGACCAAAATCACCTATTTTTACTGTGAGGTCTTCATGAAGAAATATAT__TTAGCTAGACCAAAATCACCTATTTTTACTGTGAGGTCTTCATGAAGAAATATATTAGCTAGACCAAAATCACCTATTTTTACTGTGAGGTCTTCATGAAGAAATATAT			
BRAF	p.A598_T599insV	c.1794_1795insGTT	7:140453140__T__TAAC			
BRAF	p.A598V	c.1793C>T	7:140453142__G__A			Yes
BRAF	p.G593D	c.1778G>A	7:140453157__C__T			
BRAF	p.G606G	c.1818G>A	7:140453117__C__T			
BRAF	p.K601del	c.1801_1803delAAA	7:140453131__ATTT__A			
BRAF	p.K601E	c.1801A>G	7:140453134__T__C		Yes	Yes
BRAF	p.PTPQQ490del	c.1467_1481delACCTACACCTCAGCA	7:140477826__CTGCTGAGGTGTAGGT__C			
BRAF	p.Q612*	c.1834C>T	7:140453101__G__A			
BRAF	p.T599_R603>I	c.1796_1809>TC	7:140453125__ATCGAGATTTCACTG__AGA			

Gene	Amino acid change	Nucleotide Change	Genomic Variant ID	Note	Variant Detected By GATK	Variant Detected By Samtools
BRAF	p.T599_V600>IAL	c.1796_1798CAG>TAGCTT	7:140453136__ACTG__AAAGCTA			
BRAF	p.T599_V600insDFGLAT	c.1798_1799ins18	7:140453136__A__ACTGTAGCTAGACCAAAAT			
BRAF	p.T599_V600insT	c.1797_1798insACA	7:140453137__C__CTGT			
BRAF	p.T599_V600insT	c.1796_1797insTAC	7:140453138__T__TGTA			
BRAF	p.T599del	c.1794_1796delTAC	7:140453138__TGTA__T			
BRAF	p.TAPTP488del	c.1462_1476delACAGCACCTACACCT	7:140477831__GAGGTGTAGGTGCTGT__G			
BRAF	p.V600_K601>E	c.1799_1801delTGA	7:140453133__TTCA__T			
BRAF	p.V600_S605>DV	c.1799_1814>ATGT	7:140453120__ACTCCATCGAGATTTCA__AACAT			
BRAF	p.V600>YM	c.1798_1798G>TACA	7:140453136__AC__ATGTA			
BRAF	p.V600E	c.1799T>A	7:140453136__A__T		Yes	Yes
BRAF	p.V600E	c.1799_1800TG>AA	7:140453134__TCA__TTT			
BRAF	p.V600E	c.1799_1800TG>AA	7:140453135__CA__TT			
BRCA1	p.V412I	c.1234G>A	17:41246314__C__T			
BRCA2	p.A2738S	c.8212G>T	13:32937551__G__T			
BRCA2	p.C1820R	c.5458T>C	13:32913950__T__C			
BRCA2	p.H2932Y	c.8794C>T	13:32953493__C__T			
BRE	p.I60L	c.178A>T	2:28152748__A__T			
BRIP1	p.G509F	c.1525_1526GG>TT	17:59861733__CC__AA			
C15orf55	p.R1108Q	c.3407G>A	15:34649616__G__A			
CACNA1D	p.G674D	c.2081G>A	3:53757947__G__A			
CACNA1D	p.W372C	c.1116G>T	3:53700562__G__T			Yes
CACNA1E	p.A37V	c.110C>T	1:181452990__C__T			
CACNA1E	p.I1623T	c.4868T>C	1:181735734__T__C			
CACNA1E	p.L2126P	c.6377T>C	1:181767534__T__C			
CACNA1E	p.S2104C	c.6311C>G	1:181767468__C__G			
CACNA1E	p.W1542R	c.4624T>C	1:181731728__T__C			
CCDC6	p.Q24*	c.70C>T	10:61666113__G__A			
CD163	p.A300V	c.899C>T	12:7649609__G__A			
CD163	p.G230R	c.688G>A	12:7651554__C__T			

Gene	Amino acid change	Nucleotide Change	Genomic Variant ID	Note	Variant Detected By GATK	Variant Detected By Samtools
CD163	p.S505L	c.1514C>T	12:7640590__G__A			
CD163	p.S610P	c.1828T>C	12:7640177__A__G			Yes
CD74	p.E278K	c.456G>A	5:149782174__C__T			
CD74	p.S183fs	c.547_548del AG	5:149784319__GCT__G			
CDH11	p.D355Y	c.1063G>T	16:65016141__C__A			
CDK12	p.E1162K	c.3484G>A	17:37682293__G__A			
CDKN1A	p.R9P	c.G26C	6:36651904__G__C			
CDKN2C	p.D67N	c.199G>A	1:51439634__G__A			
CHD2	p.S1812L	c.5435C>T	15:93567883__C__T			
CHD2	p.W1529fs	c.4585_4586i nsG	15:93552546__T__TG			
CHD3	p.K603N	c.G1809C	17:7800502__G__C			
CHD3	p.Q510H	c.1530G>C	17:7798683__G__C			
CHD3	p.R399H	c.1196G>A	17:7797853__G__A			
CHD4	p.V1492G	c.4475T>G	12:6691343__A__C			Yes
CHD6	p.N2470S	c.7409A>G	20:40033972__T__C			
CHD7	p.E741G	c.2222A>G	8:61707670__A__G			
CHEK2	p.H143Q	c.C429G	22:29121246__G__C			
CHEK2	p.L391F	c.1173G>T	22:29091784__C__A			
CHEK2	p.L462I	c.1384C>A	22:29090097__G__T			
CHEK2	p.N186S	c.A557G	22:29121000__T__C			
CHEK2	p.Y390*	c.1170C>G	22:29091787__G__C			
CHN1	p.I336T	c.1007T>C	2:175673728__A__G			
CLTCL1	p.A381T	c.1141G>A	22:19222058__C__T			Yes
CLTCL1	p.K1163R	c.3488A>G	22:19195776__T__C			Yes
CLTCL1	p.L1376F	c.4126C>T	22:19183842__G__A			
CNKSR1	p.Q44E	c.130C>G	1:26507021__C__G			
COL11A1	p.S240I	c.719G>T	1:103496733__C__A			
COL11A2	p.G1054S	c.3160G>A	6:33139342__C__T			
COL11A2	p.G892R	c.2674G>C	6:33141287__C__G			
COL1A1	p.P147fs	c.441delC	17:48276616__CG__C			
COL1A2	p.R996S	c.2988A>T	7:94055354__A__T			Yes
COL24A1	p.R1368G	c.4102A>G	1:86250007__T__C			
COL27A1	p.G1403_spl ice	c.4207_splice	9:117052336__A__G			
COL27A1	p.K470T	c.1409A>C	9:116931244__A__C			Yes
COL2A1	p.K106E	c.316A>G	12:48391978__T__C			
COL3A1	p.P1057A	c.3169C>G	2:189871146__C__G			
COL3A1	p.P770S	c.2308C>T	2:189866147__C__T			
COL4A1	p.D499Y	c.1495G>T	13:110844602__C__A			Yes

Gene	Amino acid change	Nucleotide Change	Genomic Variant ID	Note	Variant Detected By GATK	Variant Detected By Samtools
COL4A1	p.G832R	c.2494G>A	13:110830543__C__T			
COL4A2	p.P349L	c.1046C>T	13:111099179__C__T			
COL4A2	p.Y1557fs	c.4669delT	13:111160355__CT__C			
COL4A3	p.T255M	c.764C>T	2:228118353__C__T			Yes
COL4A4	p.G1457R	c.4369G>C	2:227875182__C__G			
COL4A4	p.K169R	c.506A>G	2:227979396__T__C			Yes
COL4A5	p.G123R	c.367G>A	X:107812034__G__A			
COL4A6	p.P1041R	c.3122C>G	X:107417689__G__C			
COL5A1	p.E677*	c.2029G>T	9:137655578__G__T			
COL5A1	p.P978S	c.2932C>T	9:137690287__C__T			
COL5A1	p.R1627Q	c.4880G>A	9:137716627__G__A			
COL5A3	p.P137S	c.409C>T	19:10116500__G__A			
COL5A3	p.P1414L	c.4241C>T	19:10079134__G__A			
COL5A3	p.P276T	c.826C>A	19:10114264__G__T			
COL5A3	p.Q236E	c.706C>G	19:10114384__G__C			
COL5A3	p.R1644H	c.4931G>A	19:10071487__C__T			
COL6A2	p.A940T	c.2818G>A	21:47552224__G__A			
COL6A2	p.G403E	c.1208G>A	21:47538972__G__A			
COL6A2	p.T207M	c.620C>T	21:47532397__C__T			
COL6A3	p.Q1309H	c.3927G>T	2:238280733__C__A			Yes
COL6A3	p.R1257K	c.3770G>A	2:238280890__C__T			
COL6A6	p.R356Q	c.1067G>A	3:130284243__G__A			
CREB3L3	p.G112S	c.334G>A	19:4157169__G__A			
CREB3L3	p.G35S	c.103G>A	19:4154971__G__A			
CREB3L3	p.L299I	c.895C>A	19:4171092__C__A			
CREBBP	p.L673*	c.2018T>A	16:3828107__A__T			
CRTC2	p.S375F	c.1124C>T	1:153924016__G__A			
CSF3R	p.G415R	c.1243G>A	1:36937076__C__T			
CTCF	p.D581H	c.1741G>C	16:67663340__G__C			
CTCF	p.K593N	c.1779G>C	16:67663378__G__C			
CTNNB1	p.A39T	c.115G>A	3:41266118__G__A			
CTNNB1	p.A43V	c.128C>T	3:41266131__C__T			
CTNNB1	p.D56N	c.166G>A	3:41266169__G__A			
CTNNB1	p.D58N	c.172G>A	3:41266175__G__A			
CTNNB1	p.E54K	c.160G>A	3:41266163__G__A			
CTNNB1	p.E55K	c.163G>A	3:41266166__G__A			
CTNNB1	p.G38D	c.113G>A	3:41266116__G__A			
CTNNB1	p.H36Y	c.106C>T	3:41266109__C__T			
CTNNB1	p.K49E	c.145A>G	3:41266148__A__G			
CTNNB1	p.K49R	c.146A>G	3:41266149__A__G			

Gene	Amino acid change	Nucleotide Change	Genomic Variant ID	Note	Variant Detected By GATK	Variant Detected By Samtools
CTNNB1	p.L46V	c.136C>G	3:41266139__C__G			
CTNNB1	p.P44L	c.131C>T	3:41266134__C__T			
CTNNB1	p.P44S	c.130C>T	3:41266133__C__T			
CTNNB1	p.P52L	c.155C>T	3:41266158__C__T			
CTNNB1	p.P744T	c.2230C>A	3:41280717__C__A			
CTNNB1	p.S29P	c.85T>C	3:41266088__T__C			
CTNNB1	p.S33F	c.98C>T	3:41266101__C__T			
CTNNB1	p.S37F	c.110C>T	3:41266113__C__T			
CTNNB1	p.S45P	c.133T>C	3:41266136__T__C			
CTNNB1	p.S47N	c.140G>A	3:41266143__G__A			
CTNNB1	p.S60F	c.179C>T	3:41266182__C__T			
CTNNB1	p.T40I	c.119C>T	3:41266122__C__T			
CTNNB1	p.T41I	c.122C>T	3:41266125__C__T			
CTNNB1	p.T41S	c.122C>G	3:41266125__C__G			
CTNNB1	p.T42I	c.125C>T	3:41266128__C__T			
CTNNB1	p.V57A	c.170T>C	3:41266173__T__C			
CTNNB1	p.Y716C	c.2147A>G	3:41280634__A__G			
CUX1	p.E1065*	c.3193G>T	7:101870709__G__T			
CUX1	p.V1195L	c.G3583T	7:101877481__G__T			
DEPDC5	p.M618V	c.1852A>G	22:32215193__A__G			Yes
DICER1	p.D1810H	c.5428G>C	14:95557639__C__G			
DICER1	p.E1813G	c.5438A>G	14:95557629__T__C			
DICER1	p.R1906S	c.5718A>C	14:95556886__T__G			
DIS3	p.D319N	c.955G>A	13:73349381__C__T			
DLEC1	p.A163P	c.487G>C	3:38087109__G__C			
DNAH9	p.A1745V	c.5234C>T	17:11607602__C__T			
DNAH9	p.A2642V	c.7925C>T	17:11687720__C__T			Yes
DNAH9	p.E4470K	c.13408G>A	17:11872791__G__A			
DNAH9	p.H3930Q	c.11790C>A	17:11827171__C__A			
DNAH9	p.K3042N	c.9126G>T	17:11726231__G__T			
DNAH9	p.L1194V	c.3580C>G	17:11584043__C__G			
DNAH9	p.P3181L	c.9542C>T	17:11757354__C__T			
DNAH9	p.V299A	c.896T>C	17:11515089__T__C			
DNAH9	p.V3213I	c.9637G>A	17:11757449__G__A			
DNMT1	p.D706N	c.2116G>A	19:10262175__C__T			
DNMT1	p.V1343M	c.4027G>A	19:10249155__C__T			
DNMT3A	p.P195L	c.584C>T	2:25497865__G__A			Yes
DNMT3A	p.Q402*	c.1204C>T	2:25469564__G__A			
DNMT3A	p.W313*	c.939G>A	2:25470535__C__T			
DOK5	p.*307C	c.921A>T	20:53267018__A__T			Yes

Gene	Amino acid change	Nucleotide Change	Genomic Variant ID	Note	Variant Detected By GATK	Variant Detected By Samtools
DOK6	p.R25fs	c.73delA	18:67231728__CA__C			
DOT1L	p.G1054D	c.3161G>A	19:2222329__G__A			
EBF1	p.R381G	c.1141A>G	5:158141175__T__C			
EED	p.G35E	c.104G>A	11:85956375__G__A			
EED	p.I251V	c.751A>G	11:85977149__A__G			
EHMT2	p.R1188L	c.3563G>T	6:31847931__C__A			
EIF1AX	p.A113_splic e	c.338_splice	X:20148726__C__G		Yes	Yes
EIF1AX	p.A113_splic e	c.338_splice	X:20148725__GC__AA			
EIF1AX	p.A113V	c.C338T	X:20148725__G__A			
EIF1AX	p.G8R	c.22G>A	X:20156735__C__T			
EIF1AX	p.G9D	c.26G>A	X:20156731__C__T			
EIF1AX	p.G9R	c.25G>C	X:20156732__C__G			
EIF2S2	p.R325P	c.974G>C	20:32677564__C__G			
ELF3	p.S133F	c.398C>T	1:201981484__C__T			
ELF4	p.T565N	c.1694C>A	X:129200994__G__T			
ELL	p.V196M	c.586G>A	19:18572546__C__T			
ERBB2	p.K937R	c.2810A>G	17:37882044__A__G			Yes
ERBB3	p.H768R	c.2303A>G	12:56490857__A__G			
ERBB4	p.S1105C	c.3314C>G	2:212251745__G__C			
ERBB4	p.V1307L	c.3919G>T	2:212248348__C__A			
ERCC3	p.K557N	c.1671G>C	2:128036808__C__G			
ERG	p.E129Q	c.385G>C	21:39795356__C__G			
ESR1	p.W383*	c.1148G>A	6:152332842__G__A			
ETV1	p.P221A	c.661C>G	7:13971268__G__C			
ETV5	p.R464C	c.C1390T	3:185766571__G__A			Yes
EVPL	p.A380_splic e	c.1138_splice	17:74015142__C__G			
EVPL	p.I1127M	c.C3381G	17:74005905__G__C			
EVPL	p.Q909*	c.2725C>T	17:74006561__G__A			
EVPL	p.V1416M	c.4246G>A	17:74005040__C__T		Yes	Yes
EZH1	p.M349L	c.1045A>T	17:40865386__T__A			
EZH1	p.Y642F	c.A1925T	17:40857116__T__A			Yes
EZR	p.L104F	c.310C>T	6:159206498__G__A			
FANCD2	p.G713D	c.2138G>A	3:10106529__G__A			
FANCD2	p.M507V	c.1519A>G	3:10091163__A__G			
FANCD2	p.V670A	c.2009T>C	3:10106101__T__C			
FANCF	p.V273G	c.818T>G	11:22646539__A__C			
FANCG	p.E48fs	c.144delA	9:35079178__CT__C			
FANCG	p.W16*	c.48G>A	9:35079474__C__T			

Gene	Amino acid change	Nucleotide Change	Genomic Variant ID	Note	Variant Detected By GATK	Variant Detected By Samtools
FANCM	p.M1561T	c.4682T>C	14:45656993__T__C			
FAT1	p.V912I	c.2734G>A	4:187628248__C__T		Yes	Yes
FBXO11	p.Q72*	c.214C>T	2:48066119__G__A			
FBXO11	p.R593T	c.1778G>C	2:48040983__C__G			
FCGR2B	p.L62P	c.185T>C	1:161641233__T__C			
FGF20	p.D177G	c.530A>G	8:16850687__T__C			
FGF7	p.W156*	c.467G>A	15:49776583__G__A			
FGFR2	p.K296N	c.888G>C	10:123279544__C__G			
FH	p.Q376H	c.1128G>T	1:241665851__C__A			Yes
FIGF	p.K286Q	c.856A>C	X:15365368__T__G			
FLG	p.G585E	c.1754G>A	1:152285608__C__T			
FLG	p.T550A	c.1648A>G	1:152285714__T__C			
FLT4	p.A706T	c.2116G>A	5:180048157__C__T			
FN1	p.R534P	c.1601G>C	2:216285470__C__G			
FNTB	p.E41fs	c.121_122ins A	14:65453792__G__GA			
GATAD2 B	p.P537L	c.1610C>T	1:153784245__G__A			
GHR	p.R386H	c.1157G>A	5:42718766__G__A			
GMPS	p.V219M	c.655G>A	3:155628609__G__A			
GNAS	p.E52K	c.154G>A	20:57428474__G__A			
GNAS	p.G251R	c.751G>C	20:57429071__G__C			
GNAS	p.Q870H	c.2610G>T	20:57484597__G__T		Yes	
GNG5	p.Q37*	c.109C>T	1:84967626__G__A			
GNGT1	p.E66Q	c.196G>C	7:93540201__G__C			
GOLGA5	p.I703M	c.2109A>G	14:93303788__A__G			
GPR98	p.D4314N	c.12940G>A	5:90074772__G__A			
GPR98	p.E4815fs	c.14444delA	5:90087089__GA__G			
GPR98	p.G2801R	c.8401G>A	5:90001231__G__A			
GPR98	p.K4611R	c.13832A>G	5:90084066__A__G			
GPR98	p.R285C	c.853C>T	5:89923208__C__T			
GYS2	p.E71Q	c.211G>C	12:21733368__C__G			
HDAC5	p.P322A	c.964C>G	17:42169608__G__C			
HDAC8	p.H334Y	c.1000C>T	X:71681859__G__A			
HIST1H1 E	p.R25H	c.74G>A	6:26156692__G__A			
HOOK3	p.A715T	c.2143G>A	8:42873627__G__A			Yes
HOOK3	p.S232C	c.695C>G	8:42819533__C__G			
HRAS	p.A11S	c.31G>T	11:534292__C__A			Yes
HRAS	p.G12_G13i nsAG	c.37_38insCC GGCG	11:534285__C__CCGCCG G			

Gene	Amino acid change	Nucleotide Change	Genomic Variant ID	Note	Variant Detected By GATK	Variant Detected By Samtools
HRAS	p.G12A	c.35G>C	11:534288__C__G			
HRAS	p.G12C	c.34G>T	11:534289__C__A			
HRAS	p.G12D	c.35G>A	11:534288__C__T			Yes
HRAS	p.G12R	c.34G>C	11:534289__C__G			
HRAS	p.G12S	c.34G>A	11:534289__C__T			
HRAS	p.G12V	c.35G>T	11:534288__C__A			
HRAS	p.G13C	c.37G>T	11:534286__C__A			
HRAS	p.G13D	c.38G>A	11:534285__C__T			
HRAS	p.G13R	c.37G>C	11:534286__C__G		Yes	Yes
HRAS	p.G13V	c.38G>T	11:534285__C__A			
HRAS	p.G15S	c.43G>A	11:534280__C__T			
HRAS	p.M72I	c.216G>C	11:533840__C__G			
HRAS	p.Q22*	c.64C>T	11:534259__G__A			
HRAS	p.Q61H	c.183G>C	11:533873__C__G			
HRAS	p.Q61K	c.181C>A	11:533875__G__T		Yes	Yes
HRAS	p.Q61K	c.181C>M	11:533875__G__A			Yes
HRAS	p.Q61K	c.181C>M	11:533875__G__C			
HRAS	p.Q61L	c.182A>T	11:533874__T__A			
HRAS	p.Q61P	c.182A>C	11:533874__T__G			
HRAS	p.Q61R	c.182A>G	11:533874__T__C		Yes	Yes
HRAS	p.S17G	c.49A>G	11:534274__T__C			
HRAS	p.V14V	c.42G>A	11:534281__C__T			
HSP90AA 1	p.D500N	c.1498G>A	14:102549628__C__T			
HSP90B1	p.K561R	c.1682A>G	12:104336889__A__G			
IFNAR2	p.K310N	c.930G>C	21:34635187__G__C			
IGF1R	p.P327H	c.980C>A	15:99440012__C__A			
IGSF1	p.I314F	c.940A>T	X:130416966__T__A			
IGSF1	p.P764R	c.2291C>G	X:130411859__G__C			
IL2RA	p.R206H	c.617G>A	10:6061871__C__T			
IL2RG	p.P361S	c.1081C>T	X:70327615__G__A			
IL7R	p.R267M	c.G800T	5:35874644__G__T			
IL7R	p.R291T	c.872G>C	5:35875685__G__C			
IL7R	p.S360G	c.1078A>G	5:35876286__A__G			
INHBC	p.R245G	c.733A>G	12:57843479__A__G			
IRF4	p.S435N	c.1304G>A	6:407546__G__A			
IRS1	p.M664I	c.1992G>T	2:227661463__C__A			Yes
ITGA10	p.R613Q	c.1838G>A	1:145534935__G__A			
ITGA3	p.D790N	c.2368G>A	17:48156258__G__A			
ITGA3	p.R874L	c.2621G>T	17:48156836__G__T			
ITGA7	p.K441T	c.1322A>C	12:56091578__T__G			

Gene	Amino acid change	Nucleotide Change	Genomic Variant ID	Note	Variant Detected By GATK	Variant Detected By Samtools
ITGA8	p.P485L	c.1454C>T	10:15655758__G__A			
ITGAV	p.P993T	c.2977C>A	2:187541588__C__A			
ITGB1	p.E546K	c.1636G>A	10:33200886__C__T			
ITGB1	p.P194R	c.581C>G	10:33215004__G__C			
ITGB3	p.E338K	c.1012G>A	17:45367119__G__A			
ITGB8	p.E43A	c.A128C	7:20403260__A__C			
ITPR2	p.A1528V	c.4583C>T	12:26731693__G__A			
ITPR2	p.A2351V	c.7052C>T	12:26572040__G__A			
ITPR2	p.E975V	c.2924A>T	12:26784809__T__A			Yes
ITPR2	p.R780H	c.2339G>A	12:26809335__C__T			
ITPR2	p.V483I	c.1447G>A	12:26818947__C__T			
JAK1	p.V764I	c.2290G>A	1:65309860__C__T			
JUN	p.S17L	c.50C>T	1:59248693__G__A			
KAT2A	p.G505S	c.1513G>A	17:40269530__C__T			
KDM2A	p.P536L	c.1607C>T	11:67012703__C__T			
KDM2A	p.S348fs	c.1043delC	11:66995592__TC__T			
KDM2B	p.T1266N	c.3797C>A	12:121877692__G__T			
KDM3A	p.I747V	c.2239A>G	2:86705781__A__G			
KDM3B	p.P780fs	c.2340delG	5:137727660__CG__C			
KDM4C	p.G302E	c.905G>A	9:6893216__G__A			
KDM5A	p.K1162Q	c.3484A>C	12:417066__T__G			
KDM6A	p.K768E	c.A2302G	X:44929202__A__G			
KDM6B	p.A884V	c.2651C>T	17:7752257__C__T			Yes
KDM6B	p.S399T	c.1196G>C	17:7750709__G__C			Yes
KDR	p.E759K	c.2275G>A	4:55964962__C__T			
KDR	p.M1285T	c.3854T>C	4:55946325__A__G			
KEAP1	p.G477S	c.1429G>A	19:10600426__C__T			
KEL	p.Q357H	c.1071G>C	7:142650897__C__G			
KIAA154 9	p.E968K	c.2902G>A	7:138597183__C__T			
KRAS	p.E31Q	c.91G>C	12:25398228__C__G			
KRAS	p.E63K	c.187G>A	12:25380271__C__T			
KRAS	p.G12A	c.35G>C	12:25398284__C__G			
KRAS	p.G12C	c.34G>T	12:25398285__C__A			
KRAS	p.G12D	c.35G>A	12:25398284__C__T		Yes	Yes
KRAS	p.G12R	c.34G>C	12:25398285__C__G		Yes	Yes
KRAS	p.G12S	c.34G>A	12:25398285__C__T			
KRAS	p.G12V	c.35G>T	12:25398284__C__A			
KRAS	p.G13A	c.38G>C	12:25398281__C__G			
KRAS	p.G13C	c.37G>T	12:25398282__C__A			
KRAS	p.G13D	c.38_39GC>A	12:25398279__CGC__CAT			

Gene	Amino acid change	Nucleotide Change	Genomic Variant ID	Note	Variant Detected By GATK	Variant Detected By Samtools
		T				
KRAS	p.G13D	c.38G>A	12:25398281__C__T			
KRAS	p.G13R	c.37G>C	12:25398282__C__G			
KRAS	p.G13S	c.37G>A	12:25398282__C__T			
KRAS	p.G60A	c.179G>C	12:25380279__C__G			
KRAS	p.Q61K	c.180_181TC>AA	12:25380277__GA__TT			
KRAS	p.Q61K	c.181C>A	12:25380277__G__T			
KRAS	p.Q61L	c.182A>T	12:25380276__T__A			
KRAS	p.Q61P	c.182A>C	12:25380276__T__G			
KRAS	p.Q61R	c.182A>G	12:25380276__T__C		Yes	Yes
LAMA2	p.R1408H	c.4223G>A	6:129649469__G__A			
LAMA3	p.E2157K	c.6469G>A	18:21484047__G__A			
LAMA3	p.I2721V	c.8161A>G	18:21501533__A__G			
LAMA4	p.A594E	c.1781C>A	6:112479970__G__T			
LAMA4	p.D783V	c.2348A>T	6:112469364__T__A			Yes
LAMA4	p.T317S	c.950C>G	6:112508668__G__C			
LAMA5	p.F824L	c.2472T>G	20:60910087__A__C			
LAMA5	p.Q3178E	c.9532C>G	20:60887079__G__C			
LAMB1	p.G964D	c.G2891A	7:107594163__C__T			
LAMB4	p.W105*	c.314G>A	7:107752270__C__T			
LAMC1	p.A161G	c.482C>G	1:183072526__C__G			
LAMC1	p.E1537K	c.4609G>A	1:183111704__G__A			
LAMC2	p.K1008R	c.3023A>G	1:183208652__A__G			
LAMC3	p.E455*	c.1363G>T	9:133917103__G__T			
LAMC3	p.Q781H	c.2343G>C	9:133936606__G__C			
LCK	p.I17T	c.50T>C	1:32739980__T__C			
LPAR4	p.I113V	c.337A>G	X:78010703__A__G			
LPAR6	p.E318K	c.952G>A	13:48985608__C__T			
LPP	p.E392Q	c.1174G>C	3:188426115__G__C			
LPP	p.N477S	c.1430A>G	3:188584007__A__G			
MAP3K1	p.G1311*	c.3931G>T	5:56180602__G__T			
MAP3K1	p.L1354V	c.4060C>G	5:56181836__C__G			
MBD1	p.P128S	c.382C>T	18:47803212__G__A			
MBD5	p.S906P	c.2716T>C	2:149240876__T__C			
MBD6	p.P245S	c.733C>T	12:57919484__C__T			
MBD6	p.T752A	c.2254A>G	12:57921648__A__G			Yes
MECP2	p.P127S	c.379C>T	X:153296900__G__A			Yes
MED23	p.M525V	c.1573A>G	6:131926420__T__C			
MEN1	p.L173fs	c.517delC	11:64575514__AG__A			
MET	p.E1378Q	c.G4132C	7:116436137__G__C			

Gene	Amino acid change	Nucleotide Change	Genomic Variant ID	Note	Variant Detected By GATK	Variant Detected By Samtools
MGA	p.V3021G	c.9062T>G	15:42059342__T__G			
MITF	p.N190S	c.569A>G	3:69987187__A__G			
MKL1	p.P451S	c.1351C>T	22:40815091__G__A			
MLL	p.D2718V	c.A8153T	11:118374769__A__T			
MLL	p.K1574R	c.4721A>G	11:118361935__A__G			
MLL	p.K2779E	c.A8335G	11:118374951__A__G			
MLL	p.N1653T	c.4958A>C	11:118362606__A__C			
MLL	p.N3061S	c.A9182G	11:118375798__A__G			
MLL	p.Q3621H	c.A10863C	11:118379887__A__C			
MLL	p.S3515V	c.10552_10553TC>GT	11:118377159__TC__GT			
MLL3	p.H2006Y	c.6016C>T	7:151878929__G__A			
MLL3	p.K3847N	c.11541G>C	7:151856077__C__G			
MLL3	p.Q1791*	c.5371C>T	7:151879574__G__A			
MLL3	p.Q3591*	c.10771C>T	7:151859891__G__A			
MLL3	p.S1955F	c.5864C>T	7:151879081__G__A			
MLL3	p.S2184C	c.6551C>G	7:151878394__G__C			
MLL3	p.S3213L	c.9638C>T	7:151864343__G__A			
MLL4	p.A1510T	c.4528G>A	19:36219029__G__A			
MLL4	p.P621fs	c.1863_1864insC	19:36212112__T__TC			
MLL5	p.D186_splice	c.557_splice	7:104715089__G__T			
MLLT1	p.N115S	c.344A>G	19:6230657__T__C			
MLLT4	p.Q1446E	c.4336C>G	6:168352394__C__G			
MN1	p.533_534QQ>Q	c.1599_1601delACA	22:28194930__CTGT__C			
MPO	p.P545fs	c.1635delC	17:56350265__TG__T			
MSH2	p.R383L	c.1148G>T	2:47656952__G__T			
MSI2	p.A268V	c.803C>T	17:55752345__C__T			Yes
MUC17	p.A1590D	c.4769C>A	7:100679466__C__A			
MUC17	p.A1703T	c.5107G>A	7:100679804__G__A			
MUC17	p.N303S	c.908A>G	7:100675605__A__G			
MUC17	p.P3155S	c.9463C>T	7:100684160__C__T			
MXRA5	p.D438A	c.1313A>C	X:3242413__T__G			
MYC	p.L348P	c.1043T>C	8:128752882__T__C			
MYST4	p.E999Q	c.2995G>C	10:76781017__G__C			
MYST4	p.L154R	c.461T>G	10:76603076__T__G			
NCOR2	p.L1651Q	c.4949T>A	12:124827559__A__T			
NF1	p.E244_splice	c.730_splice	17:29508804__G__T			
NF1	p.K1568R	c.4703A>G	17:29588854__A__G			

Gene	Amino acid change	Nucleotide Change	Genomic Variant ID	Note	Variant Detected By GATK	Variant Detected By Samtools
NF2	p.V146I	c.436G>A	22:30038263__G__A			Yes
NGF	p.K153R	c.458A>G	1:115828959__T__C			
NIN	p.G2027R	c.6079G>A	14:51192784__C__T			
NKX2-1	p.K163E	c.A487G	14:36987112__T__C			
NKX2-1	p.K25N	c.G75C	14:36988488__C__G			
TCH2	p.D1921A	c.5762A>C	1:120461954__T__G			Yes
NPRL2	p.E151K	c.451G>A	3:50386439__C__T			
NRAS	p.G12C	c.34G>T	1:115258748__C__A			
NRAS	p.G12D	c.35G>A	1:115258747__C__T			
NRAS	p.G12V	c.35G>T	1:115258747__C__A			
NRAS	p.G13A	c.38G>C	1:115258744__C__G			
NRAS	p.G13C	c.37G>T	1:115258745__C__A			
NRAS	p.G13D	c.38G>A	1:115258744__C__T			
NRAS	p.Q61E	c.181C>G	1:115256530__G__C			
NRAS	p.Q61H	c.183A>C	1:115256528__T__G			
NRAS	p.Q61H	c.183A>T	1:115256528__T__A			
NRAS	p.Q61K	c.181C>A	1:115256530__G__T		Yes	Yes
NRAS	p.Q61L	c.182A>T	1:115256529__T__A			
NRAS	p.Q61R	c.182A>G	1:115256529__T__C		Yes	Yes
NRAS	p.Q61R	c.181_182CA>AG	1:115256528__TTG__TCT			
NSD1	p.I2394fs	c.7182_7183insA	5:176721551__T__TA			
NSD1	p.K513R	c.1538A>G	5:176636938__A__G			
NSD1	p.N1488S	c.4463A>G	5:176673763__A__G			
NTN4	p.D111E	c.333T>G	12:96180969__A__C			
NTRK3	p.N294T	c.881A>C	15:88679156__T__G			
NUMA1	p.H74R	c.221A>G	11:71734181__T__C			
NUP210L	p.S1557T	c.4669T>A	1:153984831__A__T			
NUP214	p.A187T	c.559G>A	9:134004831__G__A			
NUP214	p.N1350S	c.4049A>G	9:134072930__A__G			
OBSCN	p.A1436T	c.4306G>A	1:228437938__G__A			
OBSCN	p.E1373K	c.4117G>A	1:228437749__G__A			
OBSCN	p.R4748L	c.14243G>T	1:228506696__G__T			
OBSCN	p.V2449M	c.7345G>A	1:228467094__G__A			
OBSCN	p.W1841*	c.5522G>A	1:228461984__G__A			
OSMR	p.D692G	c.2075A>G	5:38925336__A__G			
OTUD4	p.T909I	c.2726C>T	4:146059006__G__A			
PAK3	p.E197D	c.654A>T	X:110406220__A__T			Yes
PCGF2	p.G22E	c.65_66GG>AA	17:36896590__CC__TT			

Gene	Amino acid change	Nucleotide Change	Genomic Variant ID	Note	Variant Detected By GATK	Variant Detected By Samtools
PCGF2	p.R64W	c.190C>T	17:36895858__G__A			
PCK2	p.I268V	c.802A>G	14:24568395__A__G			
PCM1	p.F1253C	c.3758T>G	8:17830011__T__G			
PCSK7	p.N334S	c.1001A>G	11:117094847__T__C			
PDE4DIP	p.E2276K	c.6826G>A	1:144854644__C__T			
PDE4DIP	p.Q2060K	c.6178C>A	1:144859906__G__T			
PDE4DIP	p.Q849R	c.2546A>G	1:144906087__T__C			
PDGFRB	p.G805S	c.2413G>A	5:149500817__C__T			
PHF6	p.G93C	c.277G>T	X:133527567__G__T			
PHLPP2	p.R1177G	c.3529A>G	16:71683236__T__C			Yes
PIK3AP1	p.T171M	c.512C>T	10:98416610__G__A			
PIK3CA	p.D1056G	c.3167A>G	3:178952112__A__G			
PIK3CA	p.D538G	c.1613A>G	3:178936071__A__G			
PIK3CA	p.E542K	c.1624G>A	3:178936082__G__A			
PIK3CA	p.E545A	c.1634A>C	3:178936092__A__C			
PIK3CA	p.E545K	c.1633G>A	3:178936091__G__A			
PIK3CA	p.E545Q	c.1633G>C	3:178936091__G__C			
PIK3CA	p.E982G	c.2945A>G	3:178951890__A__G			Yes
PIK3CA	p.E982K	c.2944G>A	3:178951889__G__A			
PIK3CA	p.F1039L	c.3115T>C	3:178952060__T__C			
PIK3CA	p.G1049R	c.3145G>C	3:178952090__G__C			
PIK3CA	p.G1049S	c.3145G>A	3:178952090__G__A			
PIK3CA	p.G1050D	c.3149G>A	3:178952094__G__A			
PIK3CA	p.G118D	c.G353A	3:178917478__G__A			
PIK3CA	p.H1047L	c.3140A>T	3:178952085__A__T			
PIK3CA	p.H1047R	c.3140A>G	3:178952085__A__G			
PIK3CA	p.K1030E	c.3088A>G	3:178952033__A__G			
PIK3CA	p.K1041N	c.3123A>T	3:178952068__A__T			
PIK3CA	p.M1043I	c.3129G>C	3:178952074__G__C			
PIK3CA	p.M1043I	c.3129G>T	3:178952074__G__T			
PIK3CA	p.P539R	c.1616C>G	3:178936074__C__G			
PIK3CA	p.T1025A	c.3073A>G	3:178952018__A__G			
PIK3CA	p.T1025T	c.3075C>T	3:178952020__C__T		Yes	Yes
PIK3CA	p.T1031I	c.3092C>T	3:178952037__C__T			
PIK3CA	p.T1052I	c.3155C>T	3:178952100__C__T			
PIK3CB	p.D1067V	c.3200A>T	3:138374244__T__A			
PIK3CD	p.R821H	c.2462G>A	1:9783218__G__A			
PIK3CG	p.H471Q	c.1413C>A	7:106509419__C__A			Yes
PIK3R1	p.P617R	c.1850C>G	5:67592034__C__G			
PIK3R5	p.D76N	c.226G>A	17:8809033__C__T			

Gene	Amino acid change	Nucleotide Change	Genomic Variant ID	Note	Variant Detected By GATK	Variant Detected By Samtools
PIK3R5	p.K104E	c.310A>G	17:8808196__T__C			
PIK3R5	p.Y291fs	c.872_873ins A	17:8792478__G__GT			
PKHD1	p.H3131Y	c.9391C>T	6:51613023__G__A			
PKHD1	p.L1190F	c.3568C>T	6:51892687__G__A			
PKHD1	p.R2891C	c.8671C>T	6:51619708__G__A			
PKHD1	p.S1066L	c.3197C>T	6:51900420__G__A			
PKN2	p.D643G	c.1928A>G	1:89273120__A__G			Yes
PLAG1	p.P457S	c.1369C>T	8:57078936__G__A			
PMS1	p.S803G	c.2407A>G	2:190732589__A__G			
POLE	p.E1715Q	c.5143G>C	12:133218793__C__G			
POU2AF1	p.P93L	c.278C>T	11:111228348__G__A			
POU2F2	p.Q159H	c.477G>T	19:42603703__C__A			Yes
PPM1D	p.C478fs	c.1433_1454d elGCGCTAAAG CCCTGACTTTA AG	17:58740527__TGCGCTAA AGCCCTGACTTTAAG__T			
PPM1D	p.D470N	c.1408G>A	17:58740503__G__A			
PPM1D	p.L482fs	c.1445_1449d elTGACT	17:58740539__CTGACT__ C			
PPM1D	p.N574fs	c.1722_1723d elCT	17:58740816__ACT__A			
PPM1D	p.T406fs	c.1217delC	17:58734158__AC__A			
PPP2R1A	p.A273V	c.818C>T	19:52719042__C__T			
PPP2R1A	p.M291K	c.872T>A	19:52719096__T__A			
PPP2R3B	p.G99D	c.296G>A	X:347131__C__T			
PPP2R5C	p.A15V	c.44C>T	14:102276323__C__T			Yes
PPP2R5D	p.Q342*	c.1024C>T	6:42976211__C__T			
PPP6C	p.S208T	c.623G>C	9:127915858__C__G			
PRDM16	p.DLDS624d el	c.1870_1881d elGACCTGGAC AGC	1:3328630__GGACCTGGA CAGC__G			
PRDM16	p.P55L	c.164C>T	1:3102815__C__T			
PTCH1	p.A507V	c.1520C>T	9:98239123__G__A			
PTCH1	p.T778M	c.2333C>T	9:98229625__G__A			
PTEN	p.C124S	c.370T>A	10:89692886__T__A			
PTEN	p.D116G	c.347A>G	10:89692863__A__G			
PTEN	p.D252V	c.755A>T	10:89717730__A__T			
PTEN	p.F243S	c.728T>C	10:89717703__A__G			
PTEN	p.G129R	c.385G>A	10:89692901__G__A			
PTEN	p.H118R	c.353A>G	10:89692869__A__G			
PTEN	p.L139*	c.416T>A	10:89692932__T__A			

Gene	Amino acid change	Nucleotide Change	Genomic Variant ID	Note	Variant Detected By GATK	Variant Detected By Samtools
PTEN	p.L318F	c.952C>T	10:89720801__C__T			
PTEN	p.N323fs*21	c.968delA	10:89720816__AA__A			
PTEN	p.P339S	c.1015C>T	10:89720864__C__T			
PTEN	p.R130*	c.388C>T	10:89692904__C__T			
PTEN	p.R130Q	c.389G>A	10:89692905__G__A			
PTEN	p.R234Q	c.701G>A	10:89717676__G__A			
PTEN	p.V166I	c.496G>A	10:89711878__G__A			
PYGO1	p.H144Y	c.430C>T	15:55839051__G__A			
RAPGEF1	p.K1011R	c.3032A>G	9:134455701__T__C			
RAPGEF1	p.S148N	c.443G>A	9:134518626__C__T			
RARA	p.I258fs	c.773_774ins A	17:38508725__T__TA			
RASA1	p.G875A	c.2624G>C	5:86676346__G__C			
RASAL1	A401T	G1201A	12:113550066__C__T			
RASAL1	F474L	C1422A	12:113545983__G__T			
RASAL1	N344S	A1031G	12:113553042__T__C			
RASAL1	P385S	C1153T	12:113552632__G__A			
RASAL1	P435S	C1303T	12:113549961__G__A			
RASAL1	R438C	C1312T	12:113549953__G__A			
RASAL1	R438H	G1313A	12:113549952__C__T			
RASAL1	W594X	G1782A	12:113543564__C__T			
RASGRF2	p.R1070H	c.3209G>A	5:80508237__G__A			Yes
RASGRF2	p.V229M	c.685G>A	5:80369069__G__A			
RB1	p.P827S	c.2479C>T	13:49039494__C__T			
RB1	p.R798W	c.2392C>T	13:49039407__C__T			
RBBP4	p.E57K	c.169G>A	1:33123032__G__A			
RBL2	p.P802R	c.2405C>G	16:53504454__C__G			
RBM15	p.S212R	c.636C>G	1:110882663__C__G			
RCOR1	p.L146_splic e	c.436_splice	14:103148316__G__T			
RELN	p.L1816I	c.5446C>A	7:103202062__G__T			
RET	p.A876V	c.2627C>T	10:43615548__C__T			
RET	p.A883F	c.2646_2648A GC>TTT	10:43615566__TAGC__TT TT			
RET	p.A883F	c.2647_2648G C>TT	10:43615567__AGC__ATT			
RET	p.A883P	c.2647G>C	10:43615568__C__G			
RET	p.A883S	c.2647G>T	10:43615568__G__T			
RET	p.A883T	c.2647G>A	10:43615568__C__T			
RET	p.A919V	c.2756C>T	10:43617419__C__T			
RET	p.C611R	c.1831T>A	10:43609075__A__T			

Gene	Amino acid change	Nucleotide Change	Genomic Variant ID	Note	Variant Detected By GATK	Variant Detected By Samtools
RET	p.C611R	c.1831T>C	10:43609075__A__G			
RET	p.C618R	c.1852T>C	10:43609096__T__C			
RET	p.C618S	c.1852T>A	10:43609096__A__T			
RET	p.C618S	c.1853G>C	10:43609097__C__G			
RET	p.C618Y	c.1853G>A	10:43609097__G__A			
RET	p.C620R	c.1858T>C	10:43609102__T__C		Yes	Yes
RET	p.C620S	c.1859G>C	10:43609103__G__C			
RET	p.C620W	c.1860C>G	10:43609104__G__C			
RET	p.C620W	c.1860T>G	10:43609104__A__C			
RET	p.C630A	c.1888_1889TG>GC	10:43609936__AC__CG			
RET	p.C630G	c.1888T>G	10:43609936__T__G			
RET	p.C630R	c.1888T>C	10:43609936__T__C			
RET	p.C630S	c.1888T>A	10:43609936__T__A			
RET	p.C634A	c.1900_1901TG>GC	10:43609948__AC__CG			
RET	p.C634C	c.1902C>T	10:43609950__C__T			
RET	p.C634F	c.1901G>T	10:43609949__G__T			Yes
RET	p.C634R	c.1900T>C	10:43609948__T__C			
RET	p.C634S	c.1900T>A	10:43609948__T__A			
RET	p.C634T	c.1900_1901TG>AC	10:43609948__AC__TG			
RET	p.C634W	c.1902C>G	10:43609950__C__G		Yes	Yes
RET	p.C634Y	c.1901G>A	10:43609949__G__A			
RET	p.D627_L633>E	c.1881_1898del18	10:43609928__ATCCACTGTGCGACGAGCT__A			
RET	p.D631_I638>A	c.1892_1914>CG	10:43609939__GACGAGCTGTGCCGCACGGTGATC__GCG			
RET	p.D631_L633>E	c.1893_1898delCGAGCT	10:43609940__ACGAGCT__A			
RET	p.D631G	c.1892A>G	10:43609940__A__G			
RET	p.D631N	c.1891G>A	10:43609939__G__A			
RET	p.D898_E901del	c.2692_2703del12	10:43615612__AGATGTTTATGAA__A			
RET	p.E615K	c.1843G>A	10:43609087__G__A			
RET	p.E632_A640>VRP	c.1895_1918>TGCGGC	10:43609942__GAGCTGTGCCGCACGGTGATCGCAG__GTGCGGC			
RET	p.E632_C634>L	c.1895_1900delAGCTGT	10:43609942__GAGCTGT__G			
RET	p.E632_L633>V	c.1895_1897delAGC	10:43609942__GAGC__G			

Gene	Amino acid change	Nucleotide Change	Genomic Variant ID	Note	Variant Detected By GATK	Variant Detected By Samtools
RET	p.E632_L633del	c.1894_1899delGAGCTG	10:43609941__CGAGCTG__C			
RET	p.E632_T636>SS	c.1894_1906>AGCT	10:43609941__CGAGCTGTGCCGCA__CAGCT			
RET	p.E632fs*6	c.1894delG	10:43609941__CG__C			
RET	p.E768D	c.2304G>C	10:43613840__G__C			
RET	p.E884K	c.2650G>A	10:43615571__G__A			
RET	p.E901K	c.2701G>A	10:43615622__G__A			
RET	p.E921K	c.2761G>A	10:43617424__G__A			
RET	p.F612_C620del	c.1834_1860del27	10:43609077__CTTCCCTGAGGAGGAGAAGTGCTTCTGC__C			
RET	p.G748C	c.2242G>T	10:43612137__G__T			Yes
RET	p.G894S	c.2680G>A	10:43615601__G__A			
RET	p.G911D	c.2732G>A	10:43617395__G__A			
RET	p.L629_D631>H	c.1886_1891delTGTGCG	10:43609933__CTGTGCG__C			
RET	p.M918T	c.2753T>C	10:43617416__T__C		Yes	Yes
RET	p.P766S	c.2296C>T	10:43613832__C__T			
RET	p.Q781R	c.2342A>G	10:43613878__T__C			
RET	p.S922P	c.2764T>C	10:43617427__A__G			
RET	p.T930M	c.2789C>T	10:43617452__G__A			
RET	p.V648I	c.1942G>A	10:43609990__G__A			
RET	p.V778V	c.2334C>T	10:43613870__C__T			
RNF2	p.L58F	c.174G>C	1:185060797__G__C			Yes
ROS1	p.P1020H	c.3059C>A	6:117686282__G__T			
RPS6KB2	p.R364W	c.1090C>T	11:67201890__C__T			
RRAGD	p.L321I	c.961C>A	6:90082246__G__T			
RSBN1L	p.V767A	c.2300T>C	7:77408244__T__C			
RUNDC2A	p.A47V	c.140C>T	16:12121194__C__T			
SACS	p.H4354L	c.13061A>T	13:23904954__T__A			Yes
SACS	p.S2824L	c.8471C>T	13:23909544__G__A			
SDC4	p.Q136R	c.407A>G	20:43959044__T__C			
SEPT6	p.Y298D	c.892T>G	X:118771054__A__C			
SETBP1	p.D61V	c.182A>T	18:42281493__A__T			
SETBP1	p.E446D	c.1338A>T	18:42530643__A__T			
SETD1A	p.N1461S	c.4382A>G	16:30991489__A__G			
SETD1A	p.P1126fs	c.3376delC	16:30990482__AC__A			
SETD2	p.E1138D	c.3414G>C	3:47162712__C__G			
SETDB1	p.E1204K	c.3610G>A	1:150936158__G__A			
SF3B1	p.M876L	c.2626A>T	2:198265531__T__A			

Gene	Amino acid change	Nucleotide Change	Genomic Variant ID	Note	Variant Detected By GATK	Variant Detected By Samtools
SF3B1	p.T7I	c.20C>T	2:198299704__G__A			
SFRS2	p.S220C	c.659C>G	17:74732250__G__C			
SHANK1	p.G1245fs	c.3734delG	19:51171482__GC__G			
SHANK1	p.M906I	c.2718G>A	19:51172499__C__T			
SHANK1	p.N820K	c.C2460A	19:51189611__G__T			
SHANK1	p.P424fs	c.1271delC	19:51207038__TG__T			
SIRT4	p.R97Q	c.290G>A	12:120741654__G__A			
SLC34A2	p.T688M	c.2063C>T	4:25678361__C__T			
SMARCA 4	p.P930L	c.2789C>T	19:11132573__C__T			
SMARCA 5	p.A823P	c.2467G>C	4:144467147__G__C			
SMARCA L1	p.F728L	c.2182T>C	2:217332707__T__C			Yes
SMARCC 2	p.L446V	c.1336C>G	12:56571852__G__C			Yes
SMARCD 3	p.R425C	c.1273C>T	7:150936733__G__A			
SMC3	p.I45V	c.133A>G	10:112335096__A__G			
SOS1	p.C441Y	c.1322G>A	2:39250247__C__T			
SOX2	p.D39fs	c.117delC	3:181430264__AC__A			
SPOP	p.P94R	c.281C>G	17:47696667__G__C		Yes	Yes
SPTA1	p.E1226K	c.3676G>A	1:158618337__C__T			
SPTA1	p.P1027L	c.3080C>T	1:158623172__G__A			
SPTA1	p.P2328T	c.6982C>A	1:158583518__G__T			
SPTA1	p.Q2378_spl ice	c.7134_splice	1:158582606__C__T			
SPTA1	p.R2330_spli ce	c.6989_splice	1:158583510__C__T			
SPTA1	p.S333F	c.998C>T	1:158646045__G__A			
SRCAP	p.G2122S	c.6364G>A	16:30744989__G__A			
SRCAP	p.G86D	c.257G>A	16:30715587__G__A			
SSX1	p.F79I	c.235T>A	X:48118021__T__A			
STAG2	p.I910R	c.2729T>G	X:123211862__T__G			
STAT5B	p.E710_splic e	c.2129_splice	17:40354774__C__G			Yes
SUFU	p.Q212*	c.634C>T	10:104353429__C__T			
SUV420H 1	p.S744R	c.2232C>G	11:67925581__G__C			
SUV420H 1	p.Y818H	c.2452T>C	11:67925361__A__G			
SYK	p.T283A	c.847_splice	9:93629413__A__G			
TBC1D12	p.A260fs	c.779delC	10:96163148__GC__G			

Gene	Amino acid change	Nucleotide Change	Genomic Variant ID	Note	Variant Detected By GATK	Variant Detected By Samtools
TBC1D7	p.E184*	c.550G>T	6:13307947__C__A			
TBC1D7	p.G213R	c.637G>A	6:13307860__C__T			
TBX3	p.Q115*	c.343C>T	12:115120663__G__A			
TCF3	p.R105Q	c.314G>A	19:1627410__C__T			
TCL1B	p.P92A	c.274C>G	14:96157184__C__G			
TCP11L2	p.V193L	c.577G>C	12:106715426__G__C			
TEK	p.P530A	c.1588C>G	9:27192585__C__G			
TEK	p.R50H	c.149G>A	9:27157925__G__A			
TERT	NA	c.228C>A	5:1295228__G__T	Not captured by platform		
TERT	NA	c.228C>T	5:1295228__G__A	Not captured by platform		
TERT	NA	c.250C>T	5:1295250__G__A	Not captured by platform		
TERT	p.R470H	c.1409G>A	5:1293592__C__T			
TERT	p.S602L	c.1805C>T	5:1280418__G__A			
TET1	p.I1429T	c.4286T>C	10:70411612__T__C			
TET3	p.H710Y	c.2128C>T	2:74300714__C__T			
TFE3	p.L120R	c.359T>G	X:48896807__A__C			
TFE3	p.R412Q	c.1235G>A	X:48888961__C__T			
TG	p.C1306S	c.3917G>C	8:133920500__G__C			
TG	p.C140G	c.418T>G	8:133883736__T__G			Yes
TG	p.C164G	c.490T>G	8:133885318__T__G			
TG	p.G1975fs	c.5925_5926insAT	8:133981764__G__GAT			
TG	p.I821fs	c.2461_2465delATTCA	8:133900512__TATTCA__T			
TG	p.K1534E	c.4600A>G	8:133935654__A__G			
TG	p.L1891fs	c.5672_5673insA	8:133978928__T__TA			
TG	p.L510fs	c.1529_1532delTGAA	8:133899145__TTGAA__T			
TG	p.M346fs	c.1037_1038insG	8:133895206__T__TG			
TG	p.Q1246P	c.3737A>C	8:133919035__A__C			
TG	p.R950Q	c.2849G>A	8:133906022__G__A			
TGFBR2	p.D222G	c.A665G	3:30713340__A__G			
THBS1	p.D1001N	c.3001G>A	15:39885603__G__A			
THBS2	p.R460C	c.1378C>T	6:169637364__G__A			
THBS3	p.N693S	c.2078A>G	1:155168008__T__C			
TLR4	p.V338A	c.1013T>C	9:120475419__T__C			
TNN	p.Y199fs	c.597delC	1:175048655__AC__A			
TNR	p.I1033N	c.3098T>A	1:175325475__A__T			
TNXB	p.G2912C	c.8734G>T	6:32021216__C__A			Yes
TNXB	p.P3398S	c.10192C>T	6:32015637__G__A			

Gene	Amino acid change	Nucleotide Change	Genomic Variant ID	Note	Variant Detected By GATK	Variant Detected By Samtools
TNXB	p.V3396M	c.10186G>A	6:32015643__C__T			
TP53	p.A159P	c.475G>C	17:7578455__C__G			
TP53	p.A161D	c.482C>A	17:7578448__G__T			
TP53	p.A161T	c.481G>A	17:7578449__C__T			
TP53	p.C242fs*5	c.722delC	17:7577558__GG__G			
TP53	p.D208fs*39	c.623delA	17:7578225__GT__G			
TP53	p.D259Y	c.775G>T	17:7577506__C__A			
TP53	p.E171fs*9	c.510_511delGG	17:7578418__TCC__T			Yes
TP53	p.E221E	c.663G>A	17:7578186__C__T			
TP53	p.E258K	c.772G>A	17:7577509__C__T			
TP53	p.E285K	c.853G>A	17:7577085__C__T			
TP53	p.E343fs	c.1028_1029delAG	17:7573997__GCT__G			
TP53	p.F212fs*4	c.633_634insN	17:7578215__A__AN			
TP53	p.G154V	c.461G>T	17:7578469__C__A			Yes
TP53	p.G266E	c.797G>A	17:7577141__C__T			
TP53	p.H178D	c.532C>G	17:7578398__G__C			
TP53	p.H178fs*69	c.529delC	17:7578400__GG__G			
TP53	p.H214D	c.640C>G	17:7578209__G__C			
TP53	p.I251T	c.752T>C	17:7577529__A__G			
TP53	p.I255S	c.764T>G	17:7577517__A__C			
TP53	p.K292R	c.875A>G	17:7577063__T__C			
TP53	p.M237I	c.711G>A	17:7577570__C__T			
TP53	p.N239S	c.716_717AC>GT	17:7577563__TGT__TAC			
TP53	p.N268H	c.802A>C	17:7577136__T__G			
TP53	p.N268S	c.803A>G	17:7577135__T__C			
TP53	p.P152L	c.455C>T	17:7578475__G__A		Yes	Yes
TP53	p.P223L	c.668C>T	17:7578181__G__A			
TP53	p.P250L	c.749C>T	17:7577532__G__A			
TP53	p.P278L	c.833C>T	17:7577105__G__A			
TP53	p.Q192*	c.574C>T	17:7578275__G__A			
TP53	p.Q317*	c.949C>T	17:7576897__G__A			
TP53	p.Q375*	c.1123C>T	17:7572986__G__A			
TP53	p.R158H	c.473G>A	17:7578457__C__T			
TP53	p.R158L	c.473G>T	17:7578457__C__A			
TP53	p.R209fs*6	c.625_626delAG	17:7578222__TCT__T			
TP53	p.R213*	c.637C>T	17:7578212__G__A			Yes
TP53	p.R248G	c.742C>G	17:7577539__G__C			

Gene	Amino acid change	Nucleotide Change	Genomic Variant ID	Note	Variant Detected By GATK	Variant Detected By Samtools
TP53	p.R248Q	c.743G>A	17:7577538__C__T		Yes	Yes
TP53	p.R248W	c.742C>T	17:7577539__G__A			
TP53	p.R273C	c.817C>T	17:7577121__G__A			
TP53	p.R273H	c.818G>A	17:7577120__C__T			
TP53	p.R282W	c.844C>T	17:7577094__G__A			
TP53	p.R283R	c.849C>T	17:7577089__G__A			
TP53	p.S241fs*7	c.720_721ins NN	17:7577560__A__ANN			
TP53	p.T231A	c.691A>G	17:7577590__T__C			
TP53	p.T256T	c.768A>G	17:7577513__T__C			
TP53	p.V272L	c.814G>T	17:7577124__C__A			
TP53BP1	p.G976W	c.G2926T	15:43738684__C__A			
TPM3	p.Y163H	c.487T>C	1:154145568__A__G			
TRIP11	p.E842K	c.2524G>A	14:92471796__C__T			
TRPM4	p.G215D	c.644G>A	19:49671841__G__A			
TRPM4	p.R1095*	c.3283C>T	19:49713617__C__T			
TRPM4	p.R706C	c.2116C>T	19:49693561__C__T			Yes
TRPM4	p.S388P	c.1162T>C	19:49684617__T__C			Yes
TRRAP	p.G1498R	c.4492G>C	7:98543388__G__C			
TRRAP	p.I333T	c.998T>C	7:98501102__T__C			
TSHR	p.A428V	c.1283C>T	14:81609685__G__A			
TSHR	p.A623F	c.1867_1868G C>TT	14:81610268__TGC__TTT			
TSHR	p.A623I	c.1867_1868G C>AT	14:81610268__TGC__TAT			
TSHR	p.A623S	c.1867G>T	14:81610269__G__T			
TSHR	p.A623V	c.1868C>T	14:81610270__C__T			
TSHR	p.A627V	c.1880C>T	14:81610282__G__A			
TSHR	p.D403delD	c.1206_1208d elCGA	14:81609607__CCGA__C			
TSHR	p.D619_T62 0>S	c.1854_1858A GATA>GT	14:81610255__AAGATA__ AGT			
TSHR	p.D619delD	c.1855_1857d elGAT	14:81610256__AGAT__A			
TSHR	p.D619G	c.1856A>G	14:81610258__A__G			
TSHR	p.D633E	c.1899C>A	14:81610301__C__A			
TSHR	p.D633E	c.1899C>G	14:81610301__C__G			
TSHR	p.D633H	c.1897G>C	14:81610299__G__C			
TSHR	p.D633Y	c.1897G>T	14:81610299__G__T		Yes	Yes
TSHR	p.F631C	c.1892T>G	14:81610294__T__G			
TSHR	p.F631I	c.1891T>A	14:81610293__T__A			
TSHR	p.F631L	c.1893C>A	14:81610295__C__A		Yes	Yes

Gene	Amino acid change	Nucleotide Change	Genomic Variant ID	Note	Variant Detected By GATK	Variant Detected By Samtools
TSHR	p.F631L	c.1891T>C	14:81610293__T__C			
TSHR	p.F631V	c.1891T>G	14:81610293__T__G			
TSHR	p.G431S	c.1291G>A	14:81609693__G__A			
TSHR	p.I486F	c.1456A>T	14:81609858__A__T		Yes	Yes
TSHR	p.I486M	c.1458C>G	14:81609860__C__G			
TSHR	p.I486N	c.1457T>A	14:81609859__A__T			
TSHR	p.I568F	c.1702A>T	14:81610104__A__T			
TSHR	p.I568T	c.1703T>C	14:81610105__T__C			
TSHR	p.I630L	c.1888A>C	14:81610290__A__C		Yes	Yes
TSHR	p.I635V	c.1903A>G	14:81610305__A__G			
TSHR	p.I640K	c.1919T>A	14:81610321__A__T			
TSHR	p.I640V	c.1918A>G	14:81610320__A__G			
TSHR	p.L512Q	c.1535T>A	14:81609937__T__A			
TSHR	p.L512R	c.1535T>G	14:81609937__T__G		Yes	Yes
TSHR	p.L629F	c.1887G>C	14:81610289__G__C			
TSHR	p.L629F	c.1887G>T	14:81610289__G__T			
TSHR	p.M453T	c.1358T>C	14:81609760__T__C		Yes	Yes
TSHR	p.P639A	c.1915C>G	14:81610317__C__G			
TSHR	p.P639S	c.1915C>T	14:81610317__C__T			
TSHR	p.S281I	c.842G>T	14:81606172__G__T			
TSHR	p.S281N	c.842G>A	14:81606172__G__A			
TSHR	p.S281T	c.842G>C	14:81606172__G__C			
TSHR	p.S425I	c.1274G>T	14:81609676__G__T			
TSHR	p.S505N	c.1514G>A	14:81609916__G__A			
TSHR	p.T632A	c.1894A>G	14:81610296__A__G			
TSHR	p.T632I	c.1895C>T	14:81610297__C__T		Yes	Yes
TSHR	p.T632I	c.1895_1896CC>TT	14:81610296__ACC__ATT			
TTL	p.S151L	c.452C>T	2:113251935__C__T			
UBR5	p.A1768V	c.5303C>T	8:103297922__G__A			
USP6	p.Y1366*	c.4098T>A	17:5076150__T__A			
USP9X	p.A1104fs	c.3311_3312insA	X:41043681__C__CA			
USP9X	p.E61*	c.181G>T	X:40988337__G__T			
USP9X	p.K1798T	c.5393A>C	X:41075213__A__C			
USP9X	p.T1535_splice	c.4603_splice	X:41058005__T__C			
VEGFA	p.E31G	c.92A>G	6:43742103__A__G			
VTI1A	p.E143_splice	c.427_splice	10:114298090__G__A		Yes	
VTN	p.G128A	c.383G>C	17:26696674__C__G			

Gene	Amino acid change	Nucleotide Change	Genomic Variant ID	Note	Variant Detected By GATK	Variant Detected By Samtools
VWF	p.H2009Q	c.6027C>A	12:6105204__G__T			Yes
WDR59	p.A78V	c.233C>T	16:74990380__G__A			
WDR59	p.L883V	c.2647T>G	16:74919593__A__C			
WHSC1L1	p.P464L	c.1391C>T	8:38187086__G__A			
WHSC1L1	p.S138L	c.413C>T	8:38205277__G__A			
WRN	p.L1178P	c.3533T>C	8:31004953__T__C			
WT1	p.R443S	c.1329G>T	11:32414222__C__A			
YWHAE	p.D157V	c.470A>T	17:1264494__T__A			
YWHAG	p.G140*	c.418G>T	7:75959220__C__A			
ZFHX3	p.A1214G	c.3641C>G	16:72845826__G__C			
ZFHX3	p.G1022D	c.3065G>A	16:72984519__C__T			
ZFHX3	p.H1571R	c.4712A>G	16:72831869__T__C			
ZFHX3	p.K2987fs	c.8960_8961insA	16:72827620__C__CT			
ZFHX3	p.L2474R	c.7421T>G	16:72829160__A__C			
ZFHX3	p.P1595A	c.4783C>G	16:72831798__G__C			
ZFHX3	p.Q2304R	c.6911A>G	16:72829670__T__C			
ZNF384	p.R337C	c.1009C>T	12:6781601__G__A			
ZNF521	p.V934M	c.2800G>A	18:22805082__C__T			
ZRANB3	p.V440A	c.1319T>C	2:136026599__A__G			
