# Supplementary material for: The diagnostic application of RNA sequencing in patients with thyroid cancer: an analysis of 851 variants and 133 fusions in 524 genes
Source: BMC Bioinformatics. 2016 Jan 11;17(Suppl 1):6. doi: 10.1186/s12859-015-0849-9 (PMC4895782; doi:10.1186/s12859-015-0849-9)
Supplement: Additional file 4: — Contingency table combining FNA and tissue results. (PDF 44 kb) [file 12859_2015_849_MOESM4_ESM.pdf]

**Supplemental Table 3.** Combined VERA-FNA and tissue samples observed to harbor genetic alterations (variants and fusions) via the GATK variant caller and the chimera fusion caller.

| Genetic Alteration | Histology Malignant (Combined Tissue and FNA, n=76) | Histology Benign (Combined Tissue and FNA, n=75) |
|---|---|---|
| Positive | 38 | 15 |
| Negative | 38 | 60 |
