# Supplementary material for: The diagnostic application of RNA sequencing in patients with thyroid cancer: an analysis of 851 variants and 133 fusions in 524 genes
Source: BMC Bioinformatics. 2016 Jan 11;17(Suppl 1):6. doi: 10.1186/s12859-015-0849-9 (PMC4895782; doi:10.1186/s12859-015-0849-9)
Supplement: Additional file 5: — Variants detected by GATK and SamTools per sample. (PDF 231 kb) [file 12859_2015_849_MOESM5_ESM.pdf]

Additional file 5. Variants detected using GATK or SamTools variant calling methods. NA, not available; ND, not detected. Abbreviations: AUS/FLUS, Atypia of Undetermined Significance/Follicular Lesion of Undetermined Significance; BFN, benign follicular nodule; Cyto B, cytology benign; Cyto M, cytology malignant; FA, follicular adenoma; FC, follicular carcinoma; FN/SFN, follicular neoplasm or suspicious for neoplasm; FVPTC, follicular variant of papillary carcinoma; HCA, Hurthle cell adenoma; HCC, Hurthle cell carcinoma; HTA, Hyalinizing trabecular adenoma; LCT, lymphocytic thyroiditis; NA, not available; ND, not detected; MTC, medullary thyroid carcinoma; NHP, nodular hyperplasia; PTC, papillary thyroid carcinoma; mPTC, micro papillary thyroid carcinoma; PTC-TCV, papillary thyroid carcinoma-tall cell variant; SFM, suspicious for malignancy; WDC-NOS, well-differentiated carcinoma-not otherwise specified.

**Sample ID**	**GATK variant**	**SAMtools variant**	**Fusion**	**Histo Label**	**Subtype**	**Cytology**
FNA_1	ND	ND	ND	Histo B	BFN	AUS/FLUS
FNA_2	ND	ND	ND	Histo B	BFN	AUS/FLUS
FNA_3	ND	COL27A1 K470T	ND	Histo B	BFN	AUS/FLUS
FNA_4	ND	ND	ND	Histo B	BFN	AUS/FLUS
FNA_5	ND	ND	ND	Histo B	BFN	AUS/FLUS
FNA_6	ND	ND	ND	Histo B	FA	AUS/FLUS
FNA_7	ND	ND	ND	Histo B	FA	AUS/FLUS
FNA_8	ND	ND	ND	Histo M	FVPTC	AUS/FLUS
FNA_9	ND	ND	ND	Histo B	NHP	AUS/FLUS
FNA_10	ND	ND	BRAF:SND1	Histo B	NHP	AUS/FLUS
FNA_11	ND	ND	ND	Histo B	NHP	AUS/FLUS
FNA_12	ND	TRPM4 R706C	ND	Histo B	NHP	AUS/FLUS
FNA_13	ND	DEPDC5 M618V, PIK3CA E982G	ND	Histo B	BFN	Cyto B
FNA_14	VTI1A E143_splice	ND	ND	Histo B	BFN	Cyto B
FNA_15	ND	ND	ND	Histo B	BFN	Cyto B
FNA_16	ND	ND	ND	Histo B	BFN	Cyto B
FNA_17	ND	ND	ND	Histo B	BFN	Cyto B
FNA_18	ND	LAMA4 D783V	ND	Histo B	BFN	Cyto B
FNA_19	ND	ND	ND	Histo B	BFN	Cyto B
FNA_20	ND	ND	ND	Histo B	BFN	Cyto B
FNA_21	ND	ND	ND	Histo B	BFN	Cyto B
FNA_22	ND	CLTCL1 K1163R	ND	Histo B	BFN	Cyto B
FNA_23	ND	ND	ND	Histo B	BFN	Cyto B

Sample ID	GATK variant	SAMtools variant	Fusion	Histo Label	Subtype	Cytology
FNA_24	ND	BMP6 R379Q	ND	Histo B	BFN	Cyto B
FNA_25	ND	ND	ND	Histo B	FA	Cyto B
FNA_26	ND	ND	ND	Histo B	FA	Cyto B
FNA_27	ND	HRAS Q61R	ND	Histo B	FA	Cyto B
FNA_28	NRAS Q61K	NRAS Q61K	ND	Histo B	FA	Cyto B
FNA_29	ND	ND	ND	Histo B	FA	Cyto B
FNA_30	TSHR F631L	TSHR F631L	ND	Histo B	FA	Cyto B
FNA_31	TSHR T632I	TSHR T632I	ND	Histo B	FA	Cyto B
FNA_32	ND	ND	ND	Histo M	FC	Cyto B
FNA_33	ND	HRAS Q61K, TSHR M453T	ND	Histo M	FVPTC	Cyto B
FNA_34	ND	ND	ND	Histo B	HCA	Cyto B
FNA_35	ND	ND	ND	Histo B	HCA	Cyto B
FNA_36	ND	ND	ND	Histo B	HCA	Cyto B
FNA_37	ND	KRAS G12D	ND	Histo M	HCC	Cyto B
FNA_38	ND	HOOK3 A715T	ND	Histo M	HCC	Cyto B
FNA_39	ND	EZH1 Y642F	ND	Histo B	LCT	Cyto B
FNA_40	ND	SACS H4354L	ND	Histo B	LCT	Cyto B
FNA_41	ND	BRAF V600E	ND	Histo M	PTC	Cyto B
FNA_42	ND	BCLAF1 N221I, NF2 V146I	ND	Histo M	WDC-NOS	Cyto B
FNA_43	BRAF V600E	BRAF V600E, PKN2 D643G	ND	Histo M	FVPTC	Cyto M
FNA_44	HRAS Q61R	HRAS Q61R	ND	Histo M	FVPTC	Cyto M
FNA_45	ND	ND	ND	Histo M	FVPTC	Cyto M
FNA_46	ND	ND	ND	Histo M	HCC	Cyto M
FNA_47	ND	ND	ND	Histo M	MTC	Cyto M
FNA_48	BRAF V600E	BRAF V600E	ND	Histo M	PTC	Cyto M
FNA_49	BRAF V600E	BRAF V600E	ND	Histo M	PTC	Cyto M
FNA_50	ND	BRAF V600E	ND	Histo M	PTC	Cyto M
FNA_51	ND	ND	ND	Histo M	PTC	Cyto M
FNA_52	BRAF V600E	BRAF V600E	ND	Histo M	PTC	Cyto M
FNA_53	ND	ND	ND	Histo M	PTC	Cyto M
FNA_54	ND	ND	ND	Histo M	PTC	Cyto M
FNA_55	ND	ND	ND	Histo M	PTC	Cyto M
FNA_56	FAT1 V912I, VTI1A E143_splice	FAT1 V912I	RET:SPECC 1L	Histo M	PTC	Cyto M
FNA_57	BRAF V600E	BRAF V600E	ND	Histo M	PTC	Cyto M
FNA_58	ND	ND	ND	Histo M	PTC	Cyto M

Sample ID	GATK variant	SAMtools variant	Fusion	Histo Label	Subtype	Cytology
FNA_59	BRAF V600E	BRAF V600E	ND	Histo M	PTC	Cyto M
FNA_60	BRAF V600E	BRAF V600E	ND	Histo M	PTC	Cyto M
FNA_61	ND	ND	ND	Histo M	PTC	Cyto M
FNA_62	BRAF V600E	BRAF V600E	ND	Histo M	PTC	Cyto M
FNA_63	ND	CACND1D W372C	NCOA4:RET	Histo M	PTC	Cyto M
FNA_64	BRAF V600E	BCOR R1261G, BRAF V600E	ND	Histo M	PTC	Cyto M
FNA_65	BRAF V600E	BRAF V600E	ND	Histo M	PTC-TCV	Cyto M
FNA_66	BRAF V600E	BRAF V600E	ND	Histo M	PTC-TCV	Cyto M
FNA_67	ND	COL4A3 T255M	ND	Histo B	BFN	FN/SFN
FNA_68	TSHR M453T	HRAS A11S, TSHR M453T	ND	Histo B	BFN	FN/SFN
FNA_69	ND	ND	ND	Histo B	BFN	FN/SFN
FNA_70	ND	ND	ND	Histo B	FA	FN/SFN
FNA_71	FAT1 V912I	FAT1 V912I	ND	Histo B	FA	FN/SFN
FNA_72	ND	COL4A3 T255M	PAX8:PPAR G	Histo M	FVPTC	FN/SFN
FNA_73	ND	ND	ND	Histo B	HCA	FN/SFN
FNA_74	ND	ND	ND	Histo B	LCT	FN/SFN
FNA_75	ND	ND	ND	Histo B	NHP	FN/SFN
FNA_76	ND	ND	ND	Histo M	PTC	FN/SFN
FNA_77	ND	ITPR2 E975V	ND	Histo M	FC	SFM
FNA_78	ND	ND	PAX8:PPAR G	Histo M	FVPTC	SFM
FNA_79	ND	CLTCL1 A381T	ND	Histo M	FVPTC	SFM
FNA_80	NRAS Q61R, TP53 R248Q	NRAS Q61R, STAT5B E710_splice, TP53 R248Q	ND	Histo M	FVPTC	SFM
FNA_81	ND	ND	ND	Histo B	HTA	SFM
FNA_82	ND	ND	ND	Histo M	PTC	SFM
FNA_83	ND	ND	ND	Histo M	PTC	SFM
FNA_84	ND	ND	ND	Histo M	PTC	SFM
FNA_85	BRAF V600E	BRAF V600E	ND	Histo M	PTC	SFM
FNA_86	ND	BRAF V600E, RET C634F	ND	Histo M	PTC	SFM
FNA_87	ND	PIK3CA E982G	ND	Histo M	PTC	SFM
FNA_88	BRAF V600E	BRAF V600E, COL4A1 D499Y, VWF H2009Q	ND	Histo M	PTC-TCV	SFM
Tissue_1	ND	ND	ND	Histo B	BFN	NA
Tissue_2	PIK3CA T1025T, TSHR D633Y	PIK3CA T1025T, TSHR D633Y	ND	Histo B	CN	NA

Sample ID	GATK variant	SAMtools variant	Fusion	Histo Label	Subtype	Cytology
Tissue_3	ND	ND	ND	Histo B	FA	NA
Tissue_4	ND	ND	ND	Histo B	FA	NA
Tissue_5	ND	ND	CCDC6:RET	Histo B	FA	NA
Tissue_6	ND	ND	ND	Histo B	FA	NA
Tissue_7	ND	ND	ND	Histo B	FA	NA
Tissue_8	ND	COL1A2 R996S	ND	Histo B	FA	NA
Tissue_9	FAT1 V912I	FAT1 V912I	ND	Histo B	FA	NA
Tissue_10	GNDS Q870H	ND	ND	Histo B	FA	NA
Tissue_11	TSHR I486F	NRAS Q61K, TSHR I486F	ND	Histo B	FA	NA
Tissue_12	ND	COL6A3 Q1309H, DNMT3A P195L, ETV5 R464C	ND	Histo B	FA	NA
Tissue_13	ND	COL4A4 K169R	ND	Histo B	FA	NA
Tissue_14	NRAS Q61R	EVPL V1416M, NRAS Q61R	ND	Histo B	FA	NA
Tissue_15	NRAS Q61K	NRAS Q61K, PPP2R5C A15V, TP53 G154V	ND	Histo B	FA	NA
Tissue_16	ND	ND	ND	Histo B	FA	NA
Tissue_17	HRAS Q61K	HRAS Q61K	ND	Histo B	FA	NA
Tissue_18	ND	ND	ND	Histo B	FA	NA
Tissue_19	TSHR D633Y	EVPL V1416M, TSHR D633Y	ND	Histo B	FA	NA
Tissue_20	ND	ND	ND	Histo B	FA	NA
Tissue_21	ND	ND	ND	Histo B	HCA	NA
Tissue_22	ND	ND	ND	Histo B	HCA	NA
Tissue_23	ND	PIK3CG H471Q	ND	Histo B	LCT	NA
Tissue_24	ND	ND	ND	Histo B	NHP	NA
Tissue_25	ND	ND	ND	Histo B	NHP	NA
Tissue_26	ND	ND	ND	Histo B	NHP	NA
Tissue_27	ND	ND	ND	Histo B	NHP	NA
Tissue_28	ND	NOTCH2 D1921A	ND	Histo B	NHP	NA
Tissue_29	ND	FH Q376H	ND	Histo B	NHP	NA
Tissue_30	ND	ND	ND	Histo B	NHP	NA
Tissue_31	ND	ND	ND	Histo B	NML	NA
Tissue_32	HRAS Q61K	HRAS Q61K, KDM6B S399T	ND	Histo M	ATC	NA
Tissue_33	ND	ND	ND	Histo M	FC	NA
Tissue_34	HRAS Q61K	HRAS Q61K	ND	Histo M	FC	NA
Tissue_35	NRAS Q61R, TSHR	NRAS Q61R, TSHR	ND	Histo M	FC	NA

Sample ID	GATK variant	SAMtools variant	Fusion	Histo Label	Subtype	Cytology
	M453T	M453T				
Tissue_36	ND	ND	ND	Histo M	FC	NA
Tissue_37	BRAF V600E	BRAF V600E	ND	Histo M	FVPTC	NA
Tissue_38	BRAF V600E	BRAF V600E	ND	Histo M	FVPTC	NA
Tissue_39	ND	TP53 G154V, TSHR I630L	ND	Histo M	FVPTC	NA
Tissue_40	ND	ND	ND	Histo M	FVPTC	NA
Tissue_41	BRAF V600E	BRAF V600E, DOK5 *307C	ND	Histo M	FVPTC	NA
Tissue_42	ND	ARID5B K1027R, DNDH9 A2642V	ND	Histo M	FVPTC	NA
Tissue_43	ND	ND	ND	Histo M	FVPTC	NA
Tissue_44	ND	ND	ND	Histo M	HCC	NA
Tissue_45	ND	ND	ND	Histo M	HCC	NA
Tissue_46	ND	ND	ND	Histo M	HCC	NA
Tissue_47	HRAS Q61R	HRAS Q61R	ND	Histo M	MTC	NA
Tissue_48	HRAS Q61R	HRAS Q61R	ND	Histo M	MTC	NA
Tissue_49	RET M918T	RET M918T, , SACS H4354L, TG C140G	ND	Histo M	MTC	NA
Tissue_50	RET M918T	COL1A2 R996S, RET M918T	ND	Histo M	MTC	NA
Tissue_51	RET C620R	KDM6B S399T, RET C620R	ND	Histo M	MTC	NA
Tissue_52	ND	TG C140G	ND	Histo M	MTC	NA
Tissue_53	ND	ND	ND	Histo M	MTC	NA
Tissue_54	RET M918T	RET M918T	ND	Histo M	MTC	NA
Tissue_55	RET M918T	RET M918T	ND	Histo M	MTC	NA
Tissue_56	HRAS G13R	ERBB2 K937R, HRAS G13R	ND	Histo M	MTC	NA
Tissue_57	KRAS Q61R	KRAS Q61R	ND	Histo M	MTC	NA
Tissue_58	ND	BRAF V600E, KDM6B S399T	ND	Histo M	PTC	NA
Tissue_59	ND	BRAF V600E	ND	Histo M	PTC	NA
Tissue_60	BRAF V600E	BRAF V600E, COL27A1 K470T	ND	Histo M	PTC	NA
Tissue_61	BRAF V600E	BRAF V600E, CLTCL1 A381T, PAK3 E197D, PHLPP2 R1177G, RET C634W	ND	Histo M	PTC	NA
Tissue_62	BRAF V600E	BRAF V600E	ND	Histo M	PTC	NA
Tissue_63	BRAF V600E	BRAF V600E	ND	Histo M	PTC-TCV	NA
Tissue_64	ND	KDM6B S399T, PHLPP2 R1177G	ND	Histo U	FN	NA

Sample ID	GATK variant	SAMtools variant	Fusion	Histo Label	Subtype	Cytology
Tissue_65	ND	ND	ND	Histo U	FN	NA
Tissue_66	ND	ND	ND	Histo U	FN	NA
Tissue_67	ND	ND	ND	Histo U	FN	NA
Tissue_68	ND	ND	ND	Histo U	FN	NA
Tissue_69	NRAS Q61K	NRAS Q61K	ND	Histo U	FN	NA
Tissue_70	ND	ND	ND	Histo U	FN	NA
Tissue_71	NRAS Q61K	NRAS Q61K	ND	Histo U	FN	NA
Tissue_72	FAT1 V912I	FAT1 V912I	ND	Histo U	FN	NA
Tissue_73	EVPL V1416M	EVPL V1416M	ND	Histo U	FN	NA
Tissue_74	ND	ARID5B K1027R, CLTCL1 A381T	ND	Histo U	FN	NA
Tissue_75	ND	ND	ND	Histo U	FN	NA
Tissue_76	ND	ND	ND	Histo U	FN	NA
Tissue_77	ND	PIK3CG H471Q	ND	Histo U	FN	NA
Tissue_78	EIF1AX A113_splice, NRAS Q61K	EIF1AX A113_splice, NRAS Q61K, PAK3 E197D	ND	Histo U	FN	NA
Tissue_79	ND	ND	PAX8:PPARG	Histo U	FN	NA
Tissue_80	ND	ND	ND	Histo U	FN	NA
Tissue_81	ND	NRAS Q61R	ND	Histo U	FN	NA
Tissue_82	NRAS Q61R	COL1A2 R996S, NRAS Q61R	ND	Histo U	FN	NA
Tissue_83	HRAS Q61K	HRAS Q61K	ND	Histo U	FN	NA
Tissue_84	ND	TP53 E171fs*9	ND	Histo U	FN	NA
Tissue_85	ND	ND	ND	Histo U	FN	NA
CLIA_1	ND	POU2F2 Q159H	ND	NA	NA	AUS/FLUS
CLIA_2	ND	ND	ND	NA	NA	AUS/FLUS
CLIA_3	ND	ND	PAX8:PPARG	NA	NA	AUS/FLUS
CLIA_4	ND	ND	ND	NA	NA	AUS/FLUS
CLIA_5	ND	ND	ND	NA	NA	AUS/FLUS
CLIA_6	ND	ND	ND	NA	NA	AUS/FLUS
CLIA_7	ND	MSI2 A268V, PHLPP2 R1177G	ND	NA	NA	AUS/FLUS
CLIA_8	ND	BMP6 R379Q, TP53 R213*	ND	NA	NA	AUS/FLUS
CLIA_9	ND	ND	PAX8:PPARG	NA	NA	AUS/FLUS
CLIA_10	ND	BRAF A598V	ND	NA	NA	AUS/FLUS
CLIA_11	ND	ND	ND	NA	NA	AUS/FLUS

Sample ID	GATK variant	SAMtools variant	Fusion	Histo Label	Subtype	Cytology
CLIA_12	ND	ND	ND	NA	NA	AUS/FLUS
CLIA_13	KRAS G12D	KRAS G12D	ND	NA	NA	AUS/FLUS
CLIA_14	ND	CLTCL1 A381T	ND	NA	NA	AUS/FLUS
CLIA_15	ND	ND	ND	NA	NA	AUS/FLUS
CLIA_16	ND	AKAP9 H2680R	ND	NA	NA	AUS/FLUS
CLIA_17	TSHR I630L	TSHR I630L	ND	NA	NA	AUS/FLUS
CLIA_18	ND	ND	ND	NA	NA	AUS/FLUS
CLIA_19	ND	ND	ND	NA	NA	AUS/FLUS
CLIA_20	ND	ND	ND	NA	NA	AUS/FLUS
CLIA_21	ND	IRS1 M664I	ND	NA	NA	AUS/FLUS
CLIA_22	ND	ND	ND	NA	NA	AUS/FLUS
CLIA_23	ND	ND	ND	NA	NA	AUS/FLUS
CLIA_24	ND	ND	ND	NA	NA	AUS/FLUS
CLIA_25	ND	ND	ND	NA	NA	AUS/FLUS
CLIA_26	ND	ND	ND	NA	NA	AUS/FLUS
CLIA_27	ND	ND	ND	NA	NA	AUS/FLUS
CLIA_28	PIK3CA T1025T	KDM6B A884V, PIK3CA T1025T	ND	NA	NA	AUS/FLUS
CLIA_29	ND	ND	ND	NA	NA	AUS/FLUS
CLIA_30	TSHR I486F	CHD4 V1492G, TSHR I486F	ND	NA	NA	AUS/FLUS
CLIA_31	ND	ND	ND	NA	NA	AUS/FLUS
CLIA_32	ND	ND	ND	NA	NA	AUS/FLUS
CLIA_33	ND	ND	ND	NA	NA	AUS/FLUS
CLIA_34	ND	ND	ND	NA	NA	AUS/FLUS
CLIA_35	ND	ND	ND	NA	NA	AUS/FLUS
CLIA_36	ND	ND	ND	NA	NA	AUS/FLUS
CLIA_37	ND	BMP6 R379Q	ND	NA	NA	AUS/FLUS
CLIA_38	ND	ND	PAX8:PPARG	NA	NA	AUS/FLUS
CLIA_39	ND	RNF2 L58F	ND	NA	NA	AUS/FLUS
CLIA_40	ND	CLTCL1 A381T, RASGRF2 R1070H	ND	NA	NA	AUS/FLUS
CLIA_41	TSHR L512R	TSHR L512R	ND	NA	NA	AUS/FLUS
CLIA_42	ND	CLTCL1 A381T, MBD6 T752A	ND	NA	NA	AUS/FLUS
CLIA_43	ND	ND	ND	NA	NA	AUS/FLUS
CLIA_44	ND	CD163 S610P, SMARCC2 L446V	ND	NA	NA	AUS/FLUS
CLIA_45	NRAS Q61R	NRAS Q61R	ND	NA	NA	AUS/FLUS

Sample ID	GATK variant	SAMtools variant	Fusion	Histo Label	Subtype	Cytology
CLIA_46	ND	ND	ND	NA	NA	AUS/FLUS
CLIA_47	ND	ND	ND	NA	NA	AUS/FLUS
CLIA_48	ND	ND	ND	NA	NA	AUS/FLUS
CLIA_49	ND	ND	ND	NA	NA	AUS/FLUS
CLIA_50	ND	ND	ND	NA	NA	AUS/FLUS
CLIA_51	ND	ND	ND	NA	NA	AUS/FLUS
CLIA_52	ND	ND	ND	NA	NA	AUS/FLUS
CLIA_53	VTI1A E143_splice	ND	ND	NA	NA	Cyto B
CLIA_54	ND	ND	ND	NA	NA	FN/SFN
CLIA_55	HRAS Q61R	HRAS Q61R	ND	NA	NA	FN/SFN
CLIA_56	TSHR M453T	TSHR M453T	ND	NA	NA	FN/SFN
CLIA_57	ND	ND	ND	NA	NA	FN/SFN
CLIA_58	ND	ND	ND	NA	NA	FN/SFN
CLIA_59	ND	ND	ND	NA	NA	FN/SFN
CLIA_60	ND	ND	ND	NA	NA	FN/SFN
CLIA_61	ND	ND	ND	NA	NA	FN/SFN
CLIA_62	ND	ND	ND	NA	NA	FN/SFN
CLIA_63	ND	ND	ND	NA	NA	FN/SFN
CLIA_64	ND	ND	ND	NA	NA	FN/SFN
CLIA_65	HRAS Q61R	HRAS Q61R	ND	NA	NA	FN/SFN
CLIA_66	ND	ND	ND	NA	NA	FN/SFN
CLIA_67	ND	KRAS Q61R, RET G748C	ND	NA	NA	FN/SFN
CLIA_68	ND	ND	ND	NA	NA	FN/SFN
CLIA_69	ND	ND	ND	NA	NA	FN/SFN
CLIA_70	PIK3CA T1025T	KRAS Q61R, PIK3CA T1025T	ND	NA	NA	FN/SFN
CLIA_71	ND	ND	ND	NA	NA	FN/SFN
CLIA_72	ND	ND	ND	NA	NA	FN/SFN
CLIA_73	ND	ND	ND	NA	NA	FN/SFN
CLIA_74	ND	ND	ND	NA	NA	FN/SFN
CLIA_75	ND	TRPM4 S388P	ND	NA	NA	FN/SFN
CLIA_76	ND	ND	ND	NA	NA	FN/SFN
CLIA_77	ND	ND	ND	NA	NA	FN/SFN
CLIA_78	ND	ND	ND	NA	NA	NA
CLIA_79	KRAS Q61R	KRAS Q61R	ND	NA	NA	NA
CLIA_80	NRAS Q61R	NRAS Q61R	ND	NA	NA	NA
CLIA_81	ND	ARID5B K1027R, COL6A3 Q1309H	ND	NA	NA	NA

Sample ID	GATK variant	SAMtools variant	Fusion	Histo Label	Subtype	Cytology
CLIA_82	ND	HRAS G12D	ND	NA	NA	NA
CLIA_83	ND	ND	ND	NA	NA	NA
CLIA_84	ND	ND	ND	NA	NA	NA
CLIA_85	ND	ND	ND	NA	NA	NA
CLIA_86	ND	ND	ND	NA	NA	NA
CLIA_87	TSHR M453T	TSHR M453T	ND	NA	NA	NA
CLIA_88	SPOP P94R	ARID5B K1027R, SPOP P94R	ND	NA	NA	NA
CLIA_89	ND	ND	ND	NA	NA	NA
CLIA_90	ND	ND	ND	NA	NA	NA
CLIA_91	ND	MECP2 P127S	ND	NA	NA	NA
CLIA_92	ND	HRAS A11S	ND	NA	NA	NA
CLIA_93	ND	PAK3 E197D	ND	NA	NA	NA
CLIA_94	ND	ND	ND	NA	NA	NA
CLIA_95	BRAF K601E	BRAF K601E	ND	NA	NA	NA
CLIA_96	ND	BRAF K601E	ND	NA	NA	NA
CLIA_97	ND	COL4A4 K169R, NRAS Q61R	ND	NA	NA	NA
CLIA_98	ND	NRAS Q61R	ND	NA	NA	NA
CLIA_99	ND	ND	ND	NA	NA	NA
CLIA_100	ND	ND	ND	NA	NA	NA
CLIA_101	TP53 P152L	TP53 P152L	ND	NA	NA	NA
CLIA_102	ND	ND	ND	NA	NA	NA
CLIA_103	ND	ND	ND	NA	NA	NA
CLIA_104	ND	COL27A1 K470T, NRAS Q61R	ND	NA	NA	NA
CLIA_105	ND	TNXB G2912C	ND	NA	NA	NA
CLIA_106	ND	EZH1 Y642F, SMARCAL1 F728L	ND	NA	NA	NA
CLIA_107	ND	ND	ND	NA	NA	NA
CLIA_108	ND	PAK3 E197D	ND	NA	NA	NA
CLIA_109	ND	ARID5B K1027R	ND	NA	NA	NA
CLIA_110	ND	ND	ND	NA	NA	SFM
