# Supplementary material for: The diagnostic application of RNA sequencing in patients with thyroid cancer: an analysis of 851 variants and 133 fusions in 524 genes
Source: BMC Bioinformatics. 2016 Jan 11;17(Suppl 1):6. doi: 10.1186/s12859-015-0849-9 (PMC4895782; doi:10.1186/s12859-015-0849-9)
Supplement: Additional file 6: — Estimated mutation rates as a function of cohort size. (JPG 96 kb) [file 12859_2015_849_MOESM6_ESM.pdf]

The diagnostic application of RNA sequencing in patients with thyroid cancer: an analysis of 851 variants and 133 fusions in 524 genes

Moraima Pagan[1], Richard T. Kloos[1], Chu-Fang Lin[1], Kevin J. Travers[1], Hajime Matsuzaki[1], Ed Y. Tom[1], Su Yeon Kim[1], Mei G. Wong[1], Andrew C. Stewart[1], Jing Huang[1], P. Sean Walsh[1], Robert J. Monroe[1] and Giulia C. Kennedy[1]

CORRESPONDING AUTHOR: Giulia C. Kennedy, Ph.D. email address: giulia@veracyte.com

Keywords: Molecular testing, variants, fusions, RNA seq, genomic alterations, thyroid nodules, FNA

Abstract

Background. Thyroid carcinomas are known to harbor oncogenic driver mutations and advances in sequencing technology now allow the detection of these in fine needle aspiration biopsies (FNA). Recent work by The Cancer Genome Atlas (TCGA) Research Network has expanded the number of genetic alterations detected in papillary thyroid carcinomas (PTC). We sought to investigate the prevalence of these and other genetic alterations in diverse subtypes of thyroid nodules beyond PTC, including a variety of samples with benign histopathology. This is the first clinical evaluation of a large panel of TCGA-reported genomic alterations in thyroid FNAs.

Results. In FNAs, genetic alterations were detected in 19/44 malignant samples (43% sensitivity) and in 7/44 histopathology benign samples (84% specificity). Overall, after adding a cohort of tissue samples, 38/76 (50%) of histopathology malignant samples were found to harbor a genetic alteration, while 15/75 (20%) of benign samples were also mutated. The most frequently mutated malignant subtypes were medullary thyroid carcinoma (9/12, 75%) and PTC (14/30, 47%). Additionally, follicular adenoma, a benign subtype of thyroid neoplasm, was also found to

harbor mutations (12/29, 41%). Frequently mutated genes in malignant samples included BRAF (20/76, 26%) and RAS (9/76, 12%). Of the TSHR variants detected, (6/7, 86%) were in benign nodules. In a direct comparison of the same FNA also tested by an RNA-based gene expression classifier (GEC), the sensitivity of genetic alterations alone was 42%, compared to the 91% sensitivity achieved by the GEC. The specificity based only on genetic alterations was 84%, compared to 77% specificity with the GEC.

Conclusions. While the genomic landscape of all thyroid neoplasm subtypes will inevitably be elucidated, caution should be used in the early adoption of published mutations as the sole predictor of malignancy in thyroid. The largest set of such mutations known to date detects only a portion of thyroid carcinomas in preoperative FNAs in our cohort and thus is not sufficient to rule out cancer. Due to the finding that variants are also found in benign nodules, testing only GEC suspicious nodules may be helpful in avoiding false positives and altering the extent of treatment when selected mutations are found.

Introduction

With the wide adoption of next-generation sequencing (NGS) technologies, the number of genome variants and fusions detected in disease tissues has increased dramatically. The Catalog of Somatic Mutations in Cancer (COSMIC) database contains >100,000 somatic mutations found across >400,000 tumors [1]. The publicly accessible ClinVar database contains >170,000 variant submissions, and >118,000 of these are with clinical interpretations [2]. The Cancer Gene Atlas (TCGA) project has generated extensive genomic data on >11,000 cases across 34 different cancers, including thyroid cancer [3]. With the discovery of so many variants in tumor tissues,

expectations of their utility in diagnosing disease are high. In a recent statement by the American Thyroid Association [4], the suggestion is made that the expansion of gene sets informed by TCGA variant discovery "will likely improve the diagnostic accuracy of molecular analyses of thyroid cytology specimens and offer promise for personalizing surgical therapy, with the potential for cost and risk reduction in the diagnostic and therapeutic approaches to treating DTC (differentiated thyroid cancer)." However, the discovery of variants of uncertain significance (VUS), or variants found in benign conditions may temper these expectations.

We and others have studied the genomic landscape of thyroid neoplasms in the pursuit of diagnostic tools for managing patients with thyroid nodules. Several well-studied mutations in genes such as BRAF, N-, H-, K-RAS, and translocations of RET/PTC and PAX8/PPARG have been evaluated for use in the clinical decision-making of these patients. In recent years, a growing list of amino acid substitutions, deletions, insertions, frameshifts, truncations, extensions, gene-pair fusions, and other complex changes have been described in thyroid cancer [5-12]. The effort implemented by TCGA identified >500 novel somatic variants in papillary thyroid carcinoma (PTC), the most common type of thyroid cancer. While TCGA used malignant tumor vs. matched normal surgical tissue as the comparison for variant discovery and analysis, a comparison of malignant vs. benign thyroid neoplasms was not conducted, and an understanding of the frequency of these variants in benign thyroid nodules as well as their frequency in fine needle aspiration (FNA) biopsies is lacking. Here we used a deep RNA sequencing approach to test the hypothesis that diagnostic accuracy of thyroid cancer would be improved by testing a large number of TCGA and literature curated variants.

The management of thyroid nodules larger than 1 cm generally includes ultrasound-guided FNA for clinical decision-making. Evaluation of FNA by cytology leads to definitive benign or malignant results in 64% of cases [13] with both high negative predictive value (NPV) >95%, and high positive predictive value (PPV) >98% [14-17]. Thirteen percent of cases are non-diagnostic [13]. Among asymptomatic thyroid nodules, a benign cytological diagnosis typically averts the need for diagnostic surgery. However, 23% of nodules cannot be definitively diagnosed [13] and are instead grouped into one of three cytology indeterminate categories (Bethesda III, IV, V)[14] according to defined microscopic features. As most cytology indeterminate thyroid nodules are pathologically benign, consensus has arisen on the value of high sensitivity/high NPV value tests to "rule-out" cancer and safely avoid unnecessary diagnostic surgery [18].

The advent of molecular testing with an RNA gene expression classifier (GEC) to rule-out malignancy with high NPV has greatly reduced the number of diagnostic thyroid surgeries of cytology indeterminate nodules, as a majority of these are indeed benign [19-27]. Others have approached this problem using gene panels whose variants have been associated with malignancy [28, 29]. Whether these rule-in tests (associated with high specificity/high PPV) could be improved to have high sensitivity/high NPV for detecting malignancy (and thus become both a rule-in and rule-out test) has become a topic of interest. A panel measuring variants in seven genes failed to achieve the high sensitivity and NPV required to safely rule-out cancer, as more than half the cancers did not harbor an identified variant [29]. Panels measuring an increasing

number of variants have been tested, but not validated in blinded or comprehensive studies, so their ability to safely rule-out cancer has yet to be determined.

The large-scale effort employed by TCGA, which generated exome sequencing, copy number and transcriptional profiling data on a large number of surgical tumor specimens, was a landmark study in elucidating the genomic landscape of many important cancers [3]. One of those studied was PTC, the most common thyroid malignancy [5]. Other types of thyroid cancer were not analyzed, therefore we extended the TCGA effort, in part, by evaluating other subtypes of thyroid cancer and by studying clinically relevant biopsies (thyroid FNA) collected pre-operatively. Here we report for the first time the prevalence of many somatic variants reported by TCGA and others across a wide spectrum of thyroid neoplasms, including benign lesions, in both preoperative FNA and surgical tissue. We studied FNA samples that were collected as part of a prospective, multicenter, blinded cohort, subjected to expert histopathology review [19]; these samples were subsequently blinded and then tested for known somatic genetic alterations using the largest panel of variants yet described. We demonstrate that this panel of 851 somatic variants and 133 somatic fusion gene-pairs has the power to detect only a portion of thyroid carcinomas in preoperative FNAs, raising doubt that such panels can safely rule-out cancer and avoid unnecessary diagnostic surgery, as has been suggested [28, 4]. Importantly, some of these genetic alterations were also commonly found in histopathology benign tumors, suggesting that as variant panels grow in size, an increasing fraction of benign nodules will be falsely identified as cancerous unless careful curation of variants based on their frequencies in benign and malignant nodules is incorporated into clinical decision-making.

Materials and methods

Sample cohorts

FNAs were prospectively collected from 88 patients as part of a previously reported, blinded multi-center study (VERA FNA)[19], or from a multi-center consecutive series of 110 de-identified, pre-operative specimens with remnant nucleic acids archived by Veracyte's CLIA laboratory (CLIA FNA). An additional 85 post-surgical snap-frozen tissues were procured from multiple clinical centers across the US, including samples obtained from Asterand (Detroit, MI), Cureline (South San Francisco, CA), Proteogenex (Culver City, CA) and CHTN (http://www.chtn.nci.nih.gov/). All clinical protocols were approved by both central and intuition-specific investigational review boards, and when applicable patients provided written informed consent. All FNAs were blindly evaluated by a cytopathologist and were categorized according to the Bethesda System [14]. Blinded histopathology reference standard labels were determined by a panel of experts (VERA001 FNA)[19], or by blinded secondary review to confirm vendor labels (R. Monroe, tissue cohort) independent of all molecular testing. After secondary review 63/85 (74%) of tissues had histopathology truth labels, while 22 were designated as follicular neoplasm (FN) with uncertain histopathology (Histo U). CLIA FNA were categorized as GEC Benign or GEC Suspicious, according to their original Afirma GEC test result. RNA was extracted using the AllPrep Micro kit (Qiagen) per the manufacturer's instructions. Sequencing libraries were prepared and pooled in groups of 16 using 10-20 ng of RNA per the manufacturer's instructions using the TruSeq RNA Access kit, followed by sequencing with a NextSeq 500 instrument (Illumina) to an average depth of 50 million (from 25 million fragments) using 75bp paired-end reads.

Detection of genetic alterations

The genomic location of all variants and fusions was extracted directly from their source publications [6, 5, 28, 29, 7] or determined using reported gene ID, amino acid change and mutation annotation files (MAF) generated by Broad Institute, British Columbia Cancer Research Centre, or Baylor University and obtained through the TCGA public access portal. When this information was not available, variants were mapped to their genomic location using COSMIC, and/or cBioportal databases. The initial panel was comprised of 987 distinct genetic alterations, including 854 somatic variants and 133 somatic fusion gene-pairs comprising 524 genes, however 3 variants could not be captured by our platform. All genetic alterations included in the final panel (n=984) are known to result in protein level changes, including amino acid substitutions, deletions, insertions, frameshifts, truncations, extensions, and other complex changes. Copy number alterations were not measured. While this study focused exclusively on curated somatic gene alteration reports [6, 28, 7, 29, 5], it is possible that some mutations are present in nodules due to germline variation. Samples were scored positive using the variant and fusion detection pipelines described below.

Reads were aligned to the reference genome, build 37, via STAR v2.4.1b [30] using state of the art best practices for variant and fusion detection as follows; splice-aware alignments used annotations of all known splice junctions from Ensembl 75. De-duplication was carried out with picard v1.123 MarkDuplicates and aligned reads were processed in GATK v3.3. Variants were detected using the GATK Haplotype Caller[31]. Low quality calls were filtered out with GATK VariantFiltration. Fusions were detected from STAR outputs using the Bioconductor package

chimera [32] and all filtering settings turned on. Asymptotic standard logit intervals were used to calculate positive predictive values (PPV) and negative predictive values (NPV) along with their associated confidence intervals as previously described [33]. Whenever a zero occurs in a two-by-two contingency table, this method returns estimated adjusted logit intervals that are non-zero.

Results

Our analysis of genetic alterations using genome-wide transcriptome sequencing included 133 fusion pairs and 854 somatic variants. Of these, 133/133 (100%) of fusions and 851/854 (99.6%) of variants were detectable by our RNA sequencing effort. Only 3 variants could not be captured, because they are located at two genomic sites within the non-transcribed promoter region of the TERT gene. Hence, our results center on a final panel of 984 distinct genetic alterations spanning 524 genes. We measured sequencing quality metrics in this study, capturing a median of 48 million paired-end reads and 37 million aligned reads per sample, and computed the range of variants covered at various read depths (Additional file 1). We measured the median read-depth per variant across the pathology subtypes and found variation in coverage, consistent with biological variation in expression of transcripts harboring the genetic variants (Additional file 2). Given this biological variation among subtypes, it is important to assess individual level coverage. We found 93% of the 851 variant locations have at least one sample with 20-fold coverage (Additional file 1). Variants discussed in this paper were identified using standard variant calling methods (GATK) and for the sake of comparison, additional variants identified at a more sensitive

but less specific setting using another variant calling method (SamTools) are reported in additional file 3.

We evaluated an FNA cohort with surgical truth and found that 19/44 of histopathology malignant samples scored positive for at least one of the genetic alterations, corresponding to 43% sensitivity (Figure 1, Table 1). Histopathology benign FNAs scored positive in 7/44 cases, corresponding to 84% specificity (Table 1). The prevalence of malignant samples in this study cohort was 50%, resulting in estimates of NPV of 60% (53-66, CI), and PPV of 73% (56-85, CI). The performance of the molecular panel was also evaluated for each Bethesda cytology category (Table 2) using two cancer prevalences (Table 1). While the number of samples was low in many groups, in cytology malignant (Bethesda VI) where the highest proportion of mutated samples was observed, 14/24 samples had a detectable variant or fusion, corresponding to 58% sensitivity.

In the post-surgical tissue cohort, 19/32 histopathology malignant samples had a detectable genetic alteration (59% sensitivity), while 8/31 histopathology benign samples scored positive, a specificity of 74% (Figure 2). A single GNAS variant was observed to occur in a follicular adenoma. As this variant has been considered to be a marker for benign nodules, we excluded it from performance calculations to avoid falsely lowering the estimate of specificity. The frequency of malignant samples in this small cohort was 51% and its NPV was estimated at 64% (53-74, CI), while PPV was 70% (55-82, CI).

We also studied a consecutive FNA series (n=110) processed through our CLIA laboratory. This clinically representative cohort was comprised primarily of cytology indeterminate samples, and was evaluated to characterize the frequency of somatic genetic alterations in a broad, multicenter, US population (Figure 3). In CLIA FNA with AUS/FLUS cytology, (Bethesda III) 9/52 (17%) of samples scored positive for a genetic alteration, similar to FNAs with FN/SFN cytology (Bethesda IV) where 4/24 samples harbored a variant (17%). Since histopathology truth was not available for any CLIA FNA, we could not compute sensitivity, specificity, NPV or PPV.

We compared the results obtained from testing for genetic alterations with results obtained from testing with the Afirma GEC, an RNA-based molecular classifier designed to rule-out malignancy with 90% sensitivity and 94-95% NPV[19]. The test reports either a GEC Benign or GEC Suspicious result. In a subset of 86 VERA FNA samples with truth labels and both GEC and genetic alteration results available, testing for genetic alterations alone identified 42% of malignant samples, compared to the GEC, which identified 91% of the true malignant samples (Figure 4). These results are consistent with previously published reports confirming high sensitivity of the GEC[19, 34, 21-26]. In this cohort, testing for genetic alterations resulted in 84% specificity, compared to the GEC, which resulted in 77% specificity, higher than the 52% reported in a large, prospective, blinded multicenter study[19]. The proportion of detected genetic alterations was far higher in samples with GEC Suspicious results; 22/49 (45%) than those with GEC Benign results (3/37, 8%), resulting in an overall PPV of potentially >60% when cancer prevalence is 40%, as is the case for Afirma Suspicious nodules. This suggests that variant testing would have greater clinical utility on GEC Suspicious FNAs where the detection of genetic alterations may alter the extent of treatment

when selected mutations are found. Conversely, no cancer missed by the GEC had a genetic alteration, suggesting that mutational testing on this GEC Benign cohort would not have identified true malignant nodules and would therefore lack clinical utility.

We wanted to get a global view of genetic alterations in both of our cohorts. After combining FNA (n=88) and tissue (n=63) samples with truth labels, 38/76 (50%) of histopathology malignant samples were found to harbor a genetic alteration, and 15/75 (20%) of the histopathology benign samples were also mutated (Additional file 4). The most frequently mutated subtypes were MTC (9/12, 75%), PTC (14/30, 47%), and FA (12/29, 41%). Frequently mutated genes in malignant samples included BRAF 21/76 (28%), found predominantly in cytology malignant samples (Figure 1). In FNAs with a histopathology diagnoses of PTC, FVPTC or PTC-TCV, 13/37 (35%) were positive for the BRAF V600E mutation, and in surgical tissues with these same diagnoses, 7/12 (54%) harbored this mutation (Additional file 5). Also frequently mutated was TSHR, found in 6/75 (8%) of histology benign samples and only once in a malignant follicular carcinoma (Figures 1 and 2).

Given the rarity of most variants measured in this cohort, we investigated the effect of sample size and its impact on mutation rate estimation. At a true underlying mutation rate of 3%, 95% confidence intervals are large for sample sizes below 100 (Additional file 6), suggesting that larger cohorts would be required to achieve more precise estimates. However, the large proportion of malignant samples with no variant detected (50%), along with the reasonably high rate of benign nodules with a variant detected (20%) cannot be explained solely by an under-representation of these variants in a small sample size cohort. The variant detection rate observed in benign

nodules limits the performance of the panel, in particular confers a specificity penalty that limits the panel's ability to attain both high sensitivity and high specificity.

Discussion

In this study we demonstrate that interrogation of a large number of somatic genomic alterations, as reported by TCGA [5, 6] and others [28, 7], is limited in its ability to detect cancer with high sensitivity when applied to a diverse set of thyroid carcinomas. Published data from TCGA shows 59.7% of their PTC tissue cohort harbored a BRAF mutation, similar to the 54% we found for the PTC tissues in our study. Despite this similarity, extension of our study to other subtypes and to FNAs reveals that of the histopathology malignant samples, 41% of tissues and 57% of FNAs scored negative for any of the 984 genetic alterations tested. These results are consistent with the observations of others [4] noting that a panel of the 17 most common genetic alterations [29] failed to detect 53% of carcinomas with Bethesda III or IV cytology. Despite significant overlap with the mutations evaluated here and in those of other smaller panels [35, 36], a recent single-center study that was not blinded, reported both high sensitivity and high specificity measuring similar genetic alterations on FN/SFN [28] and/or AUS/FLUS samples [37]. While the difference in sensitivity may be partially explained by the measurement of very rare variants in these small cohorts, which is expected statistically to result in a wide range in performance, this would not explain the difference in specificity, as we detect many of the same variants in benign nodules. In addition, the impact of blinded versus unblinded histopathology truth labels, or local histopathology diagnostic trends among challenging neoplasms, may account for differing performance among similar genomic panels. Collectively, our results and those of other groups [38, 29, 36, 35, 39] do not provide sufficient evidence to support the notion that detection of known genetic alterations alone has sufficient power at this time to safely rule

out malignancy in all thyroid subtypes. Additional studies in larger FNA cohorts are clearly needed to evaluate further the utility of mutational testing in ruling out malignancy.

This analysis of a broad cohort of expertly diagnosed thyroid neoplasms establishes for the first time that recent TCGA-reported oncogenic driver alterations are commonly found in nodules with benign histopathology. While we recognize that our sample cohorts are of modest size and that more accurate mutation-detection methods may be developed and applied in the future, the overall mutation frequencies observed in histopathology benign FNA (16%) and benign tissues (26%) suggest that the use of panels with increasingly larger numbers of discovered variants may not help rule out malignancy in thyroid nodules. Without proper curation, such panels may increase the frequency of variant detection in benign nodules and generate false positive results. Many markers detected in the current study have been reported to vary widely in their individual specificity to detect malignancy [39, 29, 40]. Unfortunately, variants with high specificity such as BRAF V600E or RET/PTC fusions are the exception rather than the rule. Somatic genetic alterations such as those found in NRAS, HRAS, and PAX/PPARG are known to occur in benign nodules [35, 41-43]. The long-term significance of these mutations in benign nodules has not been fully studied [44], and some have suggested that these represent pre-malignant lesions [45, 46, 36, 35, 29]. Despite this suggestion, no clinical evidence exists to demonstrate that today's mutation-positive, but histopathology-benign tumors have a clinically meaningful rate of developing into cancer, or a higher rate of malignant transformation than mutation-negative benign lesions [44]. Thus, no evidence exists that patients benefit from the identification and treatment of mutation-positive benign nodules. In contrast, there may be a role in identifying

variants in GEC Suspicious nodules, as these genomic alterations may alter the extent of treatment when selected mutations are found.

Because many gene variants are also detected in our histologically benign nodules, an approach whereby any mutation-positive nodule is designated as “test-positive” has the risk of artificially and erroneously raising the frequency of suspected thyroid carcinoma, thus negatively impacting patients who could have benefited from a watch-and-wait approach. An additional concern is that a number of studies in the literature use mutation status to assign a diagnosis, bypassing blinded expert review to confirm that a mutation positive nodule was indeed histopathology malignant [36, 47]. This chicken-egg conundrum can result in artificially high reported sensitivity and specificity, as truly benign nodules are declared malignant based on mutation status [36, 29, 47]. A recent report indicates that a diagnosis changes from benign to malignant in 30% of RAS mutated cases when the pathologist is aware that a mutation is present [47].

Our approach measures gene variants found only in expressed genes, and therefore has the advantage of capturing biological processes such as allele-specific gene expression and imprinting. For example, variants found in DNA may have little relevance to a disease if the gene harboring the variant is not expressed in the tissue being sampled. This could be due to general lack of expression in a particular tissue, or could be due to a parental allele-of-origin effect. Our finding that gene transcripts harboring these variants shows expression patterns that differ amongst thyroid subtypes is consistent with the power of this approach to capture potential biological processes (Additional file 2). Thus in contrast to measuring variants in DNA,

our RNA transcriptome approach allows the detection of variants and fusions that have the potential to be biologically relevant and clinically meaningful.

As some genomic variants are modestly or highly associated with thyroid malignancy, there may be value to their detection when their presence would increase the extent of initial surgery from a hemi-thyroidectomy to a total thyroidectomy to avoid the need for a second completion thyroidectomy surgery amongst those with cancer. Alternatively, in seven studies evaluating a total of 730 patients whose thyroid nodules were tested with the highly sensitive RNA-based GEC, 49% were scored as benign [34, 23, 22, 21, 24, 25]. These patients are considered for clinical follow-up in lieu of diagnostic surgery with high accuracy [26], a data-driven approach that spares unnecessary morbidity. The implementation of a genomic panel in patients destined for surgery may reduce the need for this second (completion) surgery [48], especially when positive for highly specific variants.

Conclusions

Our studies in this cohort show that evaluating thyroid nodules for genetic variants increases the risk of false positives without rescuing any GEC false negatives. Our data suggest that even when testing with a large variant panel alone, a negative mutation result does not reasonably exclude cancer. However, our data also indicates that when malignancy cannot be ruled out with variant panels, a GEC benign result can first be implemented to safely exclude patients without malignancy and patients with suspicious GEC results may then benefit from evaluation with a genomic variant panel, if this new information results in a change to clinical management. A

majority of nodules with indeterminate cytology prove benign on post-operative histopathology. Hence, tests that use genomic profiling to rule-out cancer with high sensitivity have demonstrated clinical utility in the management of cytology indeterminate nodules [19-23, 25, 24, 26].

Given the extremely low frequency of many of the variants and fusions tested here, accurate estimates of test performance will require the testing of many more patient samples and cohorts. Hopefully, carefully conducted multi-center and blinded studies, with expert pathology annotation, will be forthcoming in the near future to shed light on the mutational spectrum of both benign and malignant thyroid nodules.

Additional Files

Additional file 1: Sequencing quality metrics for 851 variants

Additional file 2: Read-depths obtained per variant and per subtype

Additional file 3: Variants included in the panel (n=851)

Additional file 4: Contingency table combining FNA and tissue results

Additional file 5: Variants detected by GATK and SamTools per sample

Additional file 6: Estimated mutation rates as a function of cohort size

Abbreviations: ATA, American Thyroid Association; ATC, anaplastic thyroid carcinoma; AUS/FLUS, Atypia of Undetermined Significance/Follicular Lesion of Undetermined Significance; BFN, benign follicular nodule; CHTN, Cooperative Human Tissue Network; CLIA, Clinical Laboratory Improvement Amendments; CN, colloid nodule; COSMIC, Catalogue of Somatic Mutations in

Cancer; FA, follicular adenoma; FC, follicular carcinoma; FN, follicular neoplasm; FN/SFN, follicular neoplasm or suspicious for neoplasm; FNA, fine needle aspirate; FVPTC, follicular variant of papillary carcinoma; GEC, gene expression classifier; HCA, Hurthle cell adenoma; HCC, Hurthle cell carcinoma; HTA, Hyalinizing trabecular adenoma; LCT, lymphocytic thyroiditis; MTC, medullary thyroid carcinoma; NHP, nodular hyperplasia; NML, normal thyroid tissue; PTC, papillary thyroid carcinoma; mPTC, micro papillary thyroid carcinoma; PTC-TCV, papillary thyroid carcinoma-tall cell variant; SFM, suspicious for malignancy; TCGA, The Cancer Genome Atlas; WDC-NOS, well-differentiated carcinoma-not otherwise specified.

Competing interests: All authors are employed by Veracyte, Inc.

Authors' contributions: MP, GCK, PSW, RTK, RJM, and JH conceived of the study, participated in its design and helped draft the manuscript. KJT, YET, MGW carried out the molecular sequencing studies. CFH, HM, SYK, and ACS participated in sequence alignment and statistical analysis.

Acknowledgements: This study and the publication costs for this article were funded by Veracyte, Inc.

Authors details: [1]Veracyte, Inc., South San Francisco, CA

Declarations
This article has been published as part of *BMC Bioinformatics* Volume 17 Supplement 1, 2016: Selected articles from the Fourteenth Asia Pacific Bioinformatics Conference (APBC 2016). The full contents of the supplement are available online at http://www.biomedcentral.com/bmcbioinformatics/supplements/17/S1.

References

1. Forbes SA, Tang G, Bindal N, Bamford S, Dawson E, Cole C et al. COSMIC (the Catalogue of Somatic Mutations in Cancer): a resource to investigate acquired mutations in human cancer. Nucleic acids research. 2010;38(Database issue):D652-7. doi:10.1093/nar/gkp995.
2. Rehm HL, Berg JS, Brooks LD, Bustamante CD, Evans JP, Landrum MJ et al. ClinGen--the Clinical Genome Resource. The New England journal of medicine. 2015;372(23):2235-42. doi:10.1056/NEJMsr1406261.
3. . The Cancer Genome Atlas Research Network https://tcga-data.nci.nih.gov/tcga/tcgaHome2.jsp.
4. Ferris RL, Baloch Z, Bernet V, Chen A, Fahey TJ, 3rd, Ganly I et al. American Thyroid Association Statement on Surgical Application of Molecular Profiling for Thyroid Nodules: Current Impact on Perioperative Decision Making. Thyroid. 2015;25(7):760-8. doi:10.1089/thy.2014.0502.
5. Cancer Genome Atlas Research N. Integrated genomic characterization of papillary thyroid carcinoma. Cell. 2014;159(3):676-90. doi:10.1016/j.cell.2014.09.050.
6. Yoshihara K, Wang Q, Torres-Garcia W, Zheng S, Vegesna R, Kim H et al. The landscape and therapeutic relevance of cancer-associated transcript fusions. Oncogene. 2014. doi:10.1038/onc.2014.406.
7. Liu D, Yang C, Bojdani E, Murugan AK, Xing M. Identification of RASAL1 as a major tumor suppressor gene in thyroid cancer. J Natl Cancer Inst. 2013;105(21):1617-27. doi:10.1093/jnci/djt249.
8. Landa I, Ganly I, Chan TA, Mitsutake N, Matsuse M, Ibrahimpasic T et al. Frequent somatic TERT promoter mutations in thyroid cancer: higher prevalence in advanced forms of the disease. The Journal of clinical endocrinology and metabolism. 2013;98(9):E1562-6. doi:10.1210/jc.2013-2383.
9. Giordano TJ, Beaudenon-Huibregtse S, Shinde R, Langfield L, Vinco M, Laosinchai-Wolf W et al. Molecular testing for oncogenic gene mutations in thyroid lesions: a case-control validation study in 413 postsurgical specimens. Hum Pathol. 2014;45(7):1339-47. doi:10.1016/j.humpath.2014.03.010.
10. Kunstman JW, Juhlin CC, Goh G, Brown TC, Stenman A, Healy JM et al. Characterization of the mutational landscape of anaplastic thyroid cancer via whole-exome sequencing. Human molecular genetics. 2015;24(8):2318-29. doi:10.1093/hmg/ddu749.
11. Ganly I, Ricarte Filho J, Eng S, Ghossein R, Morris LG, Liang Y et al. Genomic dissection of Hurthle cell carcinoma reveals a unique class of thyroid malignancy. The Journal of clinical endocrinology and metabolism. 2013;98(5):E962-72. doi:10.1210/jc.2012-3539.
12. Marques AR, Espadinha C, Catarino AL, Moniz S, Pereira T, Sobrinho LG et al. Expression of PAX8-PPAR gamma 1 rearrangements in both follicular thyroid carcinomas and adenomas. The Journal of clinical endocrinology and metabolism. 2002;87(8):3947-52. doi:10.1210/jcem.87.8.8756.
13. Bongiovanni M, Spitale A, Faquin WC, Mazzucchelli L, Baloch ZW. The Bethesda System for Reporting Thyroid Cytopathology: a meta-analysis. Acta cytologica. 2012;56(4):333-9. doi:10.1159/000339959.
14. Cibas ES, Ali SZ. The Bethesda System for Reporting Thyroid Cytopathology. American journal of clinical pathology. 2009;132(5):658-65. doi:10.1309/ajcpphlwmi3jv4la.

15. Cooper DS, Doherty GM, Haugen BR, Kloos RT, Lee SL, Mandel SJ et al. Revised American Thyroid Association management guidelines for patients with thyroid nodules and differentiated thyroid cancer. Thyroid. 2009;19(11):1167-214. doi:10.1089/thy.2009.0110.
16. Yassa L, Cibas ES, Benson CB, Frates MC, Doubilet PM, Gawande AA et al. Long-term assessment of a multidisciplinary approach to thyroid nodule diagnostic evaluation. Cancer. 2007;111(6):508-16.
17. Baloch ZW, LiVolsi VA, Asa SL, Rosai J, Merino MJ, Randolph G et al. Diagnostic terminology and morphologic criteria for cytologic diagnosis of thyroid lesions: a synopsis of the National Cancer Institute Thyroid Fine-Needle Aspiration State of the Science Conference. Diagnostic cytopathology. 2008;36(6):425-37. doi:10.1002/dc.20830.
18. Xing M, Haugen BR, Schlumberger M. Progress in molecular-based management of differentiated thyroid cancer. Lancet. 2013;381(9871):1058-69. doi:10.1016/S0140-6736(13)60109-9.
19. Alexander EK, Kennedy GC, Baloch ZW, Cibas ES, Chudova D, Diggans J et al. Preoperative Diagnosis of Benign Thyroid Nodules with Indeterminate Cytology. New England Journal of Medicine. 2012;387(8):705-15. doi:10.1056/NEJMoa1203208.
20. Duick DS, Klopper J, Diggans JC, Friedman L, Kennedy GC, Lanman RB et al. The Impact of Benign Gene Expression Classifier Test Results on the Endocrinologist-Patient Decision to Operate on Patients with Thyroid Nodules with Indeterminate FNA Cytopathology. Thyroid. 2012;22(10):996-1001. doi:10.1089/thy.2012-0180.
21. Lastra RR, Pramick MR, Crammer CJ, LiVolsi VA, Baloch ZW. Implications of a suspicious afirma test result in thyroid fine-needle aspiration cytology: an institutional experience. Cancer cytopathology. 2014;122(10):737-44. doi:10.1002/cncy.21455.
22. McIver B, Castro MR, Morris JC, Bernet V, Smallridge R, Henry M et al. An independent study of a gene expression classifier (Afirma) in the evaluation of cytologically indeterminate thyroid nodules. The Journal of clinical endocrinology and metabolism. 2014;99(11):4069-77. doi:10.1210/jc.2013-3584.
23. Harrell RM, Bimston DN. Surgical utility of Afirma: effects of high cancer prevalence and oncocytic cell types in patients with indeterminate thyroid cytology. Endocrine practice : official journal of the American College of Endocrinology and the American Association of Clinical Endocrinologists. 2014;20(4):364-9. doi:10.4158/EP13330.OR.
24. Marti JL, Avadhani V, Donatelli LA, Niyogi S, Wang B, Wong RJ et al. Wide Inter-institutional Variation in Performance of a Molecular Classifier for Indeterminate Thyroid Nodules. Annals of surgical oncology. 2015. doi:10.1245/s10434-015-4486-3.
25. Sullivan PS, Hirschowitz SL, Fung PC, Apple SK. The impact of atypia/follicular lesion of undetermined significance and repeat fine-needle aspiration: 5 years before and after implementation of the Bethesda System. Cancer cytopathology. 2014;122(12):866-72. doi:10.1002/cncy.21468.
26. Witt RL. Outcome of Thyroid Gene expression Classifier Testing in Clinical Practice. Laryngoscope. 2015. doi:10.1002/lary.25607.
27. Angell TE, Frates MC, Medici M, Liu X, Kwong N, Cibas ES et al. Afirma Benign Thyroid Nodules Show Similar Growth to Cytologically Benign Nodules During Follow Up. The Journal of clinical endocrinology and metabolism. 2015:jc20152658. doi:10.1210/jc.2015-2658.
28. Nikiforov YE, Carty SE, Chiosea SI, Coyne C, Duvvuri U, Ferris RL et al. Highly accurate diagnosis of cancer in thyroid nodules with follicular neoplasm/suspicious for a follicular

neoplasm cytology by ThyroSeq v2 next-generation sequencing assay. Cancer. 2014;120(23):3627-34. doi:10.1002/cncr.29038.
29. Beaudenon-Huibregtse S, Alexander EK, Guttler RB, Hershman JM, Babu V, Blevins TC et al. Centralized molecular testing for oncogenic gene mutations complements the local cytopathologic diagnosis of thyroid nodules. Thyroid. 2014;24(10):1479-87. doi:10.1089/thy.2013.0640.
30. Dobin A, Davis CA, Schlesinger F, Drenkow J, Zaleski C, Jha S et al. STAR: ultrafast universal RNA-seq aligner. Bioinformatics (Oxford, England). 2013;29(1):15-21. doi:10.1093/bioinformatics/bts635.
31. McKenna A, Hanna M, Banks E, Sivachenko A, Cibulskis K, Kernytsky A et al. The Genome Analysis Toolkit: a MapReduce framework for analyzing next-generation DNA sequencing data. Genome Res. 2010;20(9):1297-303. doi:10.1101/gr.107524.110.
32. Beccuti M, Carrara M, Cordero F, Lazzarato F, Donatelli S, Nadalin F et al. Chimera: a Bioconductor package for secondary analysis of fusion products. Bioinformatics (Oxford, England). 2014;30(24):3556-7. doi:10.1093/bioinformatics/btu662.
33. Mercaldo ND, Lau KF, Zhou XH. Confidence intervals for predictive values with an emphasis to case-control studies. Stat Med. 2007;26(10):2170-83. doi:10.1002/sim.2677.
34. Alexander EK, Schorr M, Klopper J, Kim C, Sipos J, Nabhan F et al. Multicenter clinical experience with the Afirma gene expression classifier. The Journal of clinical endocrinology and metabolism. 2014;99(1):119-25. doi:10.1210/jc.2013-2482.
35. Nikiforov YE, Ohori NP, Hodak SP, Carty SE, LeBeau SO, Ferris RL et al. Impact of mutational testing on the diagnosis and management of patients with cytologically indeterminate thyroid nodules: a prospective analysis of 1056 FNA samples. The Journal of clinical endocrinology and metabolism. 2011;96(11):3390-7. doi:10.1210/jc.2011-1469.
36. Eszlinger M, Krogdahl A, Munz S, Rehfeld C, Precht Jensen EM, Ferraz C et al. Impact of molecular screening for point mutations and rearrangements in routine air-dried fine-needle aspiration samples of thyroid nodules. Thyroid. 2014;24(2):305-13. doi:10.1089/thy.2013.0278.
37. Nikiforov YE, Carty SE, Chiosea SI, Coyne C, Duvvuri U, Ferris RL et al. Impact of the Multi-Gene ThyroSeq Next-Generation Sequencing Assay on Cancer Diagnosis in Thyroid Nodules with Atypia of Undetermined Significance/Follicular Lesion of Undetermined Significance Cytology. Thyroid. 2015. doi:10.1089/thy.2015.0305.
38. Labourier E, Shifrin A, Busseniers AE, Lupo MA, Manganelli ML, Andruss B et al. Molecular Testing for miRNA, mRNA, and DNA on Fine-Needle Aspiration Improves the Preoperative Diagnosis of Thyroid Nodules With Indeterminate Cytology. The Journal of clinical endocrinology and metabolism. 2015;100(7):2743-50. doi:10.1210/jc.2015-1158.
39. Krane JF, Cibas ES, Alexander EK, Paschke R, Eszlinger M. Molecular analysis of residual ThinPrep material from thyroid FNAs increases diagnostic sensitivity. Cancer cytopathology. 2015;123(6):356-61. doi:10.1002/cncy.21546.
40. Nikiforov YE, Nikiforova MN, Gnepp DR, Fagin JA. Prevalence of mutations of ras and p53 in benign and malignant thyroid tumors from children exposed to radiation after the Chernobyl nuclear accident. Oncogene. 1996;13(4):687-93.
41. Esapa CT, Johnson SJ, Kendall-Taylor P, Lennard TW, Harris PE. Prevalence of Ras mutations in thyroid neoplasia. Clin Endocrinol (Oxf). 1999;50(4):529-35.
42. Raman P, Koenig RJ. Pax-8-PPAR-gamma fusion protein in thyroid carcinoma. Nature reviews Endocrinology. 2014;10(10):616-23. doi:10.1038/nrendo.2014.115.

43. Gupta N, Dasyam AK, Carty SE, Nikiforova MN, Ohori NP, Armstrong M et al. RAS mutations in thyroid FNA specimens are highly predictive of predominantly low-risk follicular-pattern cancers. The Journal of clinical endocrinology and metabolism. 2013;98(5):E914-22. doi:10.1210/jc.2012-3396.
44. Medici M, Kwong N, Angell TE, Marqusee E, Kim MI, Frates MC et al. The variable phenotype and low-risk nature of RAS-positive thyroid nodules. BMC Med. 2015;13(1):184. doi:10.1186/s12916-015-0419-z.
45. Nikiforova MN, Nikiforov YE. Molecular genetics of thyroid cancer: implications for diagnosis, treatment and prognosis. Expert Rev Mol Diagn. 2008;8(1):83-95. doi:10.1586/14737159.8.1.83.
46. Nikiforova MN, Nikiforov YE. Molecular diagnostics and predictors in thyroid cancer. Thyroid. 2009;19(12):1351-61. doi:10.1089/thy.2009.0240.
47. Eszlinger M, Piana S, Moll A, Bosenberg E, Bisagni A, Ciarrocchi A et al. Molecular testing of thyroid fine-needle aspirations improves presurgical diagnosis and supports the histologic identification of minimally invasive follicular thyroid carcinomas. Thyroid. 2015;25(4):401-9. doi:10.1089/thy.2014.0362.
48. Yip L, Wharry LI, Armstrong MJ, Silbermann A, McCoy KL, Stang MT et al. A clinical algorithm for fine-needle aspiration molecular testing effectively guides the appropriate extent of initial thyroidectomy. Ann Surg. 2014;260(1):163-8. doi:10.1097/SLA.0000000000000215.

Figure legends

Figure 1. Performance across VERA FNA data set (n=88) and genetic alterations observed by cytology (A) and histology subtype (B). The width of each band of the circos plots is proportional to the number of samples harboring a particular variant or fusion. A complete list of all variants and fusions detected can be found in Additional files 3 and 7. Abbreviations: AUS/FLUS, Atypia of Undetermined Significance/Follicular Lesion of Undetermined Significance; BFN, benign follicular nodule; Cyto B, cytology benign; Cyto M, cytology malignant; FA, follicular adenoma; FC, follicular carcinoma; FN/SFN, follicular neoplasm or suspicious for neoplasm; FVPTC, follicular variant of papillary carcinoma; HCA, Hurthle cell adenoma; HCC, Hurthle cell carcinoma; LCT, lymphocytic thyroiditis; MTC, medullary thyroid carcinoma; NHP, nodular hyperplasia; PTC, papillary thyroid carcinoma; PTC-TCV, papillary thyroid carcinoma-tall cell variant; SFM, suspicious for malignancy; WDC-NOS, well-differentiated carcinoma-not otherwise specified.

Figure 2. Performance across tissue data set (n=63) showing genetic alterations observed per subtype. Abbreviations: ATC, anaplastic thyroid carcinoma; BFN, benign follicular nodule; CN, colloid nodule; FA, follicular adenoma; FC, follicular carcinoma; FVPTC, follicular variant of papillary carcinoma; HCA, Hurthle cell adenoma; HCC, Hurthle cell carcinoma; Histo U, histology uncertain; LCT, lymphocytic thyroiditis; MTC, medullary thyroid carcinoma; NHP, nodular hyperplasia; NML, normal thyroid; PTC, papillary thyroid carcinoma; PTC-TCV, papillary thyroid carcinoma-tall cell variant; SFM, suspicious for malignancy; WDC-NOS, well-differentiated carcinoma-not otherwise specified.

Figure 3. Variants detected in CLIA FNA data set (n=110) showing genetic alterations observed per subtype. Abbreviations: AUS/FLUS, Atypia of Undetermined Significance/Follicular Lesion of Undetermined Significance; Cyto B, cytology benign; Cyto NA, cytology category not available; FN/SFN, follicular neoplasm or suspicious for neoplasm; NPA, negative percent agreement; PPA, positive percent agreement.

Figure 4. Comparison of genetic alteration test results versus an RNA gene expression classifier (GEC) in FNA samples tested with both methods (n=86). Abbreviations: Abbreviations: False Pos, false positive; False Neg, false negative; True Pos, true positive; True Neg, true negative.

Table 1. Performance comparison across sample cohorts

Performance of Genetic Alterations (Variants and Fusions)						
			Cancer Prevalence Used in Calculation			
			PPV		NPV	
**Sample Cohort**	**Sensitivity**	**Specificity**	**24%**	**40%**	**24%**	**40%**
All VERA FNA (n=88)	43% (28-59)	84% (70-93)	46% (29-65)	64% (46-79)	82% (78-86)	69% (62-75)
AUS/FLUS & FN/SFN only VERA FNA (n=22)	33% (1-91)	84% (60-97)	40% (9-82)	58% (17-90)	80% (64-90)	65% (45-81)
Tissue (n=63)	59% (41-76)	74% (55-88)	42% (27-58)	61% (44-75)	85% (78-90)	73% (63-81)
Combined VERA FNA and Tissue (n=151)	50% (38-62)	80% (69-88)	44% (32-57)	63% (50-73)	84% (80-87)	71% (65-76)

Table 2. FNA performance per cytology group

Performance on Cytology Benign (n=30)

Genetic Alteration	Histology Malignant (n=6)	Histology Benign (n=24)
Positive	0	4
Negative	6	20

Sensitivity, 0% (0-46); Specificity, 83% (63-95); PPV, 16% (5-50); NPV, 79% (73-85), prevalence of malignant lesions, 20%. Estimated performance when malignancy is prevalent at 6%: PPV, 5% (1-20); NPV, 94% (91-96).

Performance on AUS/FLUS (n=12)

Genetic Alteration	Histology Malignant (n=1)	Histology Benign (n=11)
Positive	0	1
Negative	1	10

Sensitivity, 0% (0-98); Specificity, 91% (59-100); PPV, 16% (4-45); NPV, 93% (87-97), prevalence of malignant lesions, 8%. Estimated performance when malignancy is prevalent at 24%: PPV, 40% (12-74); NPV, 81% (66-90).

Performance on FN/SFN (n=10)

Genetic Alteration	Histology Malignant (n=2)	Histology Benign (n=8)
Positive	1	2
Negative	1	6

Sensitivity, 50% (1-99); Specificity, 75% (35-97); PPV, 33% (7-76); NPV, 86% (59-96), prevalence of malignant lesions, 20%. Estimated performance when malignancy is prevalent at 24%: PPV, 39% (9-80); NPV, 83% (53-95).

Performance on SFM (n=12)

Genetic Alteration	Histology Malignant (n=11)	Histology Benign (n=1)
Positive	4	0
Negative	7	1

Sensitivity, 36% (11-69); Specificity, 100% (3-100); PPV, 92% (76-98); NPV, 9% (4-17), prevalence of malignant lesions, 92%. Estimated performance when malignancy is prevalent at 24%: PPV, 25% (8-53); NPV, 77% (58-88). Estimated performance when malignancy is prevalent at 62%: PPV, 64% (32-85); NPV, 39% (21-59).

Performance on Cytology Malignant (n=24)

Genetic Alteration	Histology Malignant (n=24)	Histology Benign (n=0)
Positive	14	0
Negative	10	0

Sensitivity, 58% (37-78); Specificity, NA% (NA); PPV, NA% (NA); NPV, NA% (NA), prevalence of malignant lesions, 100%. Estimated performance when malignancy is prevalent at 95%: PPV, NA% (NA); NPV, NA% (NA).
