# Supplementary material for: The diagnostic application of RNA sequencing in patients with thyroid cancer: an analysis of 851 variants and 133 fusions in 524 genes
Source: BMC Bioinformatics. 2016 Jan 11;17(Suppl 1):6. doi: 10.1186/s12859-015-0849-9 (PMC4895782; doi:10.1186/s12859-015-0849-9)
Supplement: Additional file 7: — Fusions tested (n=133). (PDF 51 kb) [file 12859_2015_849_MOESM7_ESM.pdf]

Additional file 7. Fusions tested (n=133)

ACACA_C20orf72	EML4_ALK	LMBRD1_LGSN	SOS1_MAP4K3
AGGF1_RAF1	ERC1_RET	LRP8_PAX8	SPECC1L_RET
AGK_BRAF	ETV6_NTRK3	MACF1_BRAF	SPSB1_H6PD
AKAP13_RET	FAM114A2_BRAF	MALAT1_ALK	SQSTM1_NTRK1
AKAP9_BRAF	FGFR2_OFD1	MKRN1_BRAF	SRPK2_PUS7
ANK3_RET	FKBP15_RET	MRPS16_TTC18	SSBP2_NTRK1
ANKRD11_DPEP1	FLJ10661_THADA	MTMR12_TERT	STRN_ALK
AP2M1_SEC63	GAS8_WDR59	MYH13_RET	TAF3_ZDHHC14
APLP2_CD40	GGNBP2_ZDHHC16	NCOA4_RET	TBL1XR1_RET
APLP2_EEA1	GOLGA5_RET	NCOR1_RHOU	TFG_ALK
APP_SEC63	GPX3_CHD4	NFIX_GATAD2A	TFG_MET
ARHGAP19_RRP12	GPX3_SEC63	NUP188_PHYHD1	TFG_NTRK1
BCKDHA_TMEM91	GPX3_SQSTM1	PAX8_GLIS1	TFG_NTRK2
BCL2L11_BRAF	GPX4_SEC63	PAX8_NFE2L2	TG_THADA
BRAF_AP3B1	GSN_AKAP13	PAX8_PPARG	THADA_IGF2BP3
BRAF_C7orf10	GSN_CTNNB1	PCM1_RET	C6orf35_PREP
BRAF_FAM114A2	GSN_HLA-DPB1	PDIA4_SIAE	TPM3_NTRK1
BRAF_CIITA	GTF2IRD1_ALK	PLVAP_ABCF1	TPR_NTRK1
BRAF_SVOPL	HLA-B_RNASEH2B	PLVAP_FOXP1	TPT1_SIAE
BRAF_ERC1	HOOK3_RET	POR_PIBF1	TRA2A_THADA
BSG_EEA1	HTATSF1_BRS3	POR_BRAF	TRIM24_RET
C3_ELK3	IFT74_CACNA2D3	PRCC_TMED5	TRIM27_RET
CCDC6_RET	IGF2_SNW1	PRKAR1A_RET	TRIM33_RET
CCNY_BRAF	IGF2R_ARFGEF2	RBM28_AOAH	UACA_LTK
CHI3L1_CBS	IGFBP5_ARHGEF4	RBPMS_NTRK3	UPF3A_CDC16
CNPY3_TPO	IGFBP5_SIAE	RNF138_RNF125	VCL_FGFR2
COL9A3_ELK3	INTS3_CHTOP	RPS2P32_THADA	VPS13B_CCDC6
CREB3L2_PPARG	IQGAP1_ZNF774	S100A6_BCL7A	WARS_CCNY
CSGALNACT2_SLC16A9	IRF2BP2_NTRK1	SCAF8_KIAA0564	ZC3HAV1_BRAF
CTSB_CBS	IYD_BIRC6	SEC16A_NOTCH1	ZDHHC14_C6orf35
CTSB_EIF4A2	JUNB_SEC63	SEPT9_KRT32	ZMYND19_ARRDC1
DNAJC6_PDE1C	KAT7_IGF2BP1	SLAMF1_CD1A	
DYNC1H1_SIAE	KIF5B_RET	SLC34A2_CLDN7	
EIF4G2_NUCB2	LLGL1_ALKBH5	SND1_BRAF	
